# Species–landscape interactions drive divergent population trajectories in four forest‐dependent Afromontane forest songbird species within a biodiversity hotspot in South Africa

**DOI:** 10.1111/eva.13306

**Published:** 2021-10-28

**Authors:** Jake M. Mulvaney, Conrad A. Matthee, Michael I. Cherry

**Affiliations:** ^1^ Department of Botany and Zoology Stellenbosch University Matieland South Africa

**Keywords:** Afromontane forest, birds, effective population size, gene flow, landscape genetics, microsatellites

## Abstract

Species confined to naturally fragmented habitats may exhibit intrinsic population complexity which may challenge interpretations of species response to anthropogenic landscape transformation. In South Africa, where native forests are naturally fragmented, forest‐dependent birds have undergone range declines since 1992, most notably among insectivores. These insectivores appear sensitive to the quality of natural matrix habitats, and it is unknown whether transformation of the landscape matrix has disrupted gene flow in these species. We undertook a landscape genetics study of four forest‐dependent insectivorous songbirds across southeast South Africa. Microsatellite data were used to conduct a priori optimization of landscape resistance surfaces (land cover, rivers and dams, and elevation) using cost‐distances along least‐cost pathway (LCP), and resistance distances (IBR). We detected pronounced declines in effective population sizes over the past two centuries for the endemic forest specialist *Cossypha dichroa* and *Batis capensis*, alongside recent gene flow disruption in *B. capensis*, *C. dichroa* and *Pogonocichla stellata*. Landscape resistance modelling showed both native forest and dense thicket configuration facilitates gene flow in *P. stellata*, *B. capensis* and *C. dichroa*. Facultative dispersal of *P. stellata* through dense thicket likely aided resilience against historic landscape transformation, whereas combined forest‐thicket degradation adversely affected the forest generalist *B. capensis*. By contrast, *Phylloscopus ruficapilla* appears least reliant upon landscape features to maintain gene flow and was least impacted by anthropogenic landscape transformation. Collectively, gene flow in all four species is improved at lower elevations, along river valleys, and riparian corridors— where native forest and dense thicket better persist. Consistent outperformance of LCP over IBR land‐cover models for *P. stellata*, *B. capensis* and *C. dichroa* demonstrates the benefits of wildlife corridors for South African forest‐dependent bird conservation, to ameliorate the extinction debts from past and present anthropogenic forest exploitation.

## 1 INTRODUCTION

Anthropogenic habitat fragmentation is a global threat to biodiversity, yet responses to this phenomenon vary across taxa (Epps & Keyghobadi, 2015; Lowe et al., 2015; Radespiel & Bruford, 2014). Species differ in sensitivity to habitat degradation following fragmentation (Amos et al., 2012; Devictor et al., 2008; Dondina et al., 2017), and landscape configuration change may independently alter local and long‐distance dispersal within a species (Freckleton et al., 2005; Richardson et al., 2016). The impacts of these environmental disturbances may take multiple generations to be detected within populations (Epps & Keyghobadi, 2015; Lowe et al., 2015; Samarasin et al., 2017), further complicating assessments of the ecological effects of distinct historic and contemporary anthropogenic activity. Interpretations of species responses to habitat fragmentation can be particularly challenging in populations confined to naturally fragmented habitats, where population complexity may arise naturally (Epps & Keyghobadi, 2015; Fenderson et al., 2020; Richardson et al., 2016). In fragmented landscapes, more vagile species better retain functional connectivity (Amos et al., 2014; Callens et al., 2011; Canales‐Delgadillo et al., 2012; DeCamargo et al., 2018; Kalle et al., 2018), as do species that facultatively disperse through otherwise unsuitable intermediary habitats (Keeley et al., 2017). This latter trait is underappreciated in landscape ecology, yet can be critical for understanding structural connectivity between spatially discrete metapopulations of vulnerable species (Driscoll et al., 2013; Kadmon & Allouche, 2007; Kupfer et al., 2006). As the loss of important matrix elements potentially impedes species dispersal, thereby exacerbating the effects of primary habitat fragmentation, the identification and preservation of these elements may prove necessary for long‐term species viability, even in cases where the matrix is infrequently utilized. Testing tolerance to both natural and anthropogenic fragmentation is best achieved by comparative research on multiple species which differ in their level of habitat specialization and mobility.

In South Africa, native forests comprise a highly fragmented biome confined to 0.5% of the country's land area (Mucina & Geldenhuys, 2006). This biome is subdivided into Afromontane forests, which are mostly scattered across low‐ and mid‐elevation slopes of inland mountains, and Indian Ocean coastal belt (IOCB) forests, which are discontinuously present along the eastern coast (von Maltitz et al., 2003; Mucina, 2018). In both sub‐biomes, forest fragmentation arose naturally through palaeoclimatic shifts (Eeley et al., 1999; Lawes et al., 2007), but has been exacerbated by anthropogenic deforestation of over 80% of IOCB forests, and 15% of Afromontane forests during the past two centuries (Berliner, 2009; Olivier et al., 2013). Commercial logging largely ceased by 1940 (Adie et al., 2013; Lawes et al., 2007), yet many forest remnants remain degraded, partly due to widespread illegal harvesting of forest products (Leaver & Cherry, 2020a), as well as the reduced structural connectivity of this biome following clearance of small forest patchworks (Kotze & Lawes, 2007), and conversion of the landscape matrix (Ehlers‐Smith et al., 2019, 2020; Freeman et al., 2018; Russell & Ward, 2016).

Anthropogenic pressures placed on South African forests have reportedly caused declines in forest‐dependent bird species, especially among insectivores (Cooper et al., 2017; Freeman et al., 2018). This group is sensitive not only to forest loss and degradation but also to conversion of the natural vegetation matrix—a trait less apparent in other South African forest‐dependent birds (Freeman et al., 2018; Neuschulz et al., 2013; Olivier & van Aarde, 2017). Clearance of coastal thicket, a vegetation type resembling low, recovering IOCB forest, is shown to impede the interforest connectivity of these bird species, as well as forest‐dependent mammals, both at the local and regional scale (Ehlers‐Smith et al., 2017a, 2017b, 2019, 2020). Despite these community‐level observations, it is unknown whether elements of the natural landscape matrix facilitate gene flow in forest‐dependent insectivorous birds, and the population genetic stability of these species remains unassessed.

Accordingly, we conducted a comparative landscape genetic study of four forest‐dependent insectivorous songbird species, focussing on the southeast region of South Africa, where ranges declines of forest‐dependent birds between 1992 and 2014 have been most substantial (Cooper et al., 2017). Our study aims were to assess contemporary levels of genetic connectivity between forest metapopulations within each species and to infer whether these species facultatively disperse through the regional landscape matrix. Additionally, we sought to evaluate the historic stability of the effective population sizes within each species. We undertook this study using microsatellite markers and employed an a priori landscape resistance modelling technique developed by Peterman et al. (2014) and Peterman (2018) to conduct our landscape genetics investigation. We hypothesized that (1) connectivity between regional forest fragments would vary between species, depending on known species vagility; (2) each species would exhibit facultative dispersal through well‐wooded habitats (thicket) to facilitate gene flow between naturally fragmented forest; and (3) species with greater forest specialization which had experienced greater contemporary range declines would have more rapidly decreasing effective population sizes.

## 2 MATERIALS AND METHODS

### 2.1 Study species

We selected four forest‐dependent insectivorous songbirds which experienced range declines across South Africa from 1992 to 2014 (Cooper et al., 2017): *Batis capensis* (−1.3% national decline); *Cossypha dichroa* (−19.5% national decline); *Phylloscopus ruficapilla* (−20.7% national decline); *Pogonocichla stellata* (−23.0% national decline). The IUCN Red List (2020) regards each species as of Least Concern, although only *B. capensis* is not in global decline (BirdLife International, 2016a, 2016b, 2017a, 2017b). These small (>50 g) species co‐occur in forests across southeast South Africa below 30°S (Hockey et al., 2005). Three species—*C. dichroa*, *P. ruficapilla* and *P. stellata*—display greater habitat specialization to Afromontane forests (Berruti, 1997; Oatley, 1997a, 1997b), whereas the fourth—*B. capensis*—extends beyond Afromontane forest into mesic and valley subtypes of Albany thicket (Johnson, 1997).

These species are not trans‐ or intracontinental migrants (Hockey et al., 2005) and would be affected only by regional anthropogenic activity; *C. dichroa* is endemic to South Africa. Within South Africa, each species may undertake seasonal altitudinal migration between inland and coastal forests (Johnson & Maclean, 1994; Oatley, 2017), especially *P. stellata* (Craig & Hulley, 2019; Oatley, 1982a), although such movements do not involve whole‐population shifts. Ad hoc South African recapture records (Oschadleus & Ranwashe, 2017) suggest higher adult vagility in *P. stellata* (83 km) and *B. capensis* (50 km) compared to *C. dichroa* (8.5km) and *P. ruficapilla* (3.4 km). The former two species more readily traverse open matrix habitats (Aben et al., 2012, 2014; Callens et al., 2011; Dane & Bolton, 2017; Galbusera et al., 2004), although seasonally migrating *P. stellata* may prefer navigating along riparian forest/thicket corridors (Oatley, 1982a, 2017).

### 2.2 Field sampling and laboratory procedures

**FIGURE 1:**
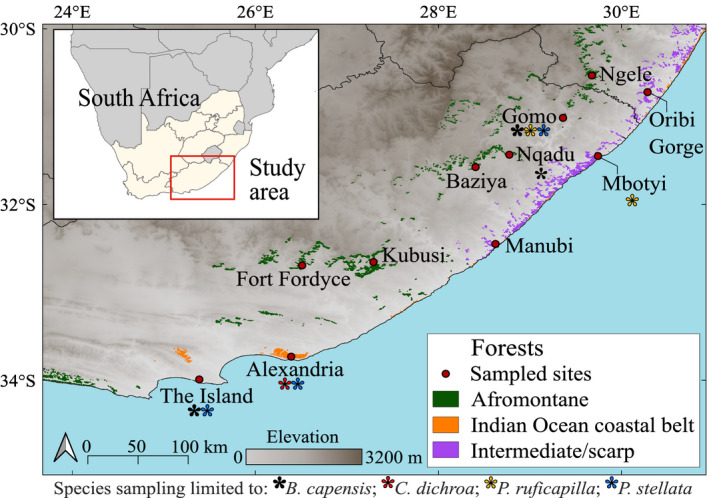
The distribution of Afromontane (green), Indian Ocean coastal belt (IOCB) (orange) and intermediate scarp (purple) forests across the Eastern Cape and southern KwaZulu‐Natal provinces of South Africa, shown alongside sampled forest locations. Coloured asterisks indicate which species of the four study species were sampled within a forest site. Forests without asterisks are represented by all four study species

From 2017 to 2018, standardized mist‐netting was conducted in six Afromontane forests, two temperate IOCB forests and three forests which are intermediate between both sub‐biomes (scarp forests) (Figure 1), visiting each forest for 3 weeks. These 11 forests in the Eastern Cape and southern KwaZulu‐Natal Provinces of South Africa fall within the Maputo‐Pondoland‐Albany biodiversity hotspot (Mittermeier et al., 2004) and were chosen to maximize equal sampling of these scarce species. In total, 114 *B. capensis*, 94 *C. dichroa*, 92 *P. ruficapilla*, and 200 *P. stellata* were captured (Table 1). Birds were banded to prevent resampling and released at the capture location after sampling of 20–50 μl of blood, collected from the brachial vein using sterile hypodermic needles and heparinized tubes, in conformity with South African legal requirements (see Acknowledgements). Collected blood samples were preserved in 500 μl 95% ethanol, and genomic DNA was extracted using a Nucleospin Tissue DNA extraction kit (Macherey‐Nagel). For each species, we screened a separate microsatellite library available from past literature (9–29 loci per species; 55 loci total) and obtained for each species a unique set of eight informative loci (see AppendixS1 for microsatellite locus screening and amplification conditions). We randomized within‐species samples prior to amplification to minimize false‐positive discovery from downstream analyses (Meirmans, 2015). Microsatellite alleles were genotyped on an ABI377xlsequencer (CAF, Stellenbosch), against LIZ 500© internal size marker, and scored in GENEIOUS 7.1.4 (©Biomatters), using three positive control individuals per species for each marker to verify scoring accuracy.

**TABLE 1:** Sample sizes, estimates of genetic diversity, and inbreeding coefficients within each forest for the four focal bird species

	Forest	*N*	AR	PrAR	*H*_o_	*H*_e_	*F*_IS_
*B. capensis*	Ngele	6	4.750	0.475	0.771	0.775	0.005
	Oribi	8	4.250	0.035	0.650	0.717	0.103
	Gomo	17	6.750	0.179	0.603	0.682	0.119
	Nqadu	8	5.625	0.243	0.607	0.806	0.260
	Baziya	14	6.500	0.253	0.580	0.688	0.161
	Manubi	16	7.625	0.148	0.578	0.705	0.184
	Kubusi	13	6.375	0.109	0.635	0.708	0.165
	Fort Fordyce	18	7.125	0.252	0.597	0.712	0.107
	The Island	14	5.625	0.070	0.607	0.720	0.162
	Total	114	10.625	0.196	0.625	0.709	0.151
*C. dichroa*	Ngele	6	4.000	0.228	0.688	0.653	−0.058
	Oribi	5	3.125	0.157	0.500	0.536	0.077
	Baziya	12	5.000	0.225	0.635	0.636	0.001
	Manubi	22	7.500	0.345	0.710	0.692	−0.027
	Kubusi	26	7.000	0.395	0.702	0.720	0.026
	Fort Fordyce	17	5.750	0.278	0.632	0.639	0.011
	Alexandria	6	3.625	0.220	0.667	0.642	−0.042
	Total	94	8.000	0.264	0.648	0.636	0.002
*P. ruficapilla*	Ngele	20	1.429	0.104	0.425	0.361	−0.184
	Oribi	6	2.250	0.005	0.325	0.364	0.119
	Mbotyi	6	2.375	0.050	0.429	0.541	−0.135
	Gomo	11	2.250	0.046	0.386	0.343	0.001
	Baziya	14	2.500	0.032	0.417	0.415	−0.003
	Manubi	15	2.875	0.100	0.367	0.401	0.087
	Kubusi	9	2.375	0.003	0.375	0.354	−0.063
	Fort Fordyce	11	0.286	0.002	0.403	0.390	−0.035
	Total	92	3.375	0.043	0.391	0.381	−0.047
*P. stellata*	Ngele	30	6.625	0.212	0.667	0.673	0.009
	Oribi	13	4.875	0.122	0.625	0.683	0.088
	Gomo	26	6.125	0.188	0.705	0.679	−0.038
	Baziya	15	5.875	0.181	0.667	0.659	−0.012
	Manubi	28	6.250	0.094	0.612	0.668	0.086
	Kubusi	26	6.250	0.176	0.606	0.652	0.072
	Fort Fordyce	39	5.875	0.058	0.622	0.648	0.042
	Alexandria	17	4.750	0.070	0.656	0.605	−0.088
	The Island	6	4.375	0.319	0.646	0.595	0.095
	Total	200	9.750	0.185	0.646	0.651	0.022

### 2.3 Population genetic diversity and structure

**FIGURE 2:**
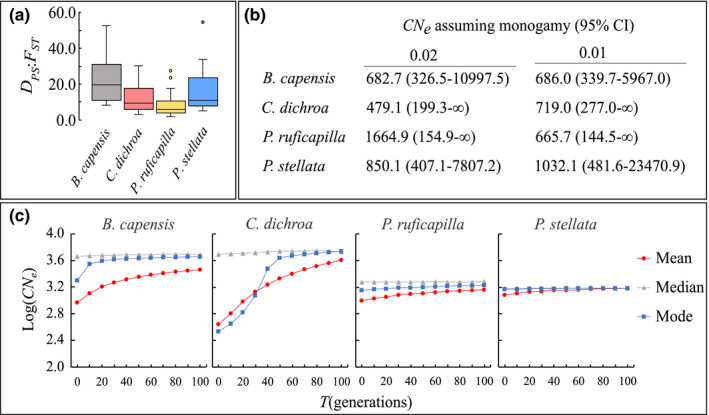
Demographic trends within *B. capensis*, *C. dichroa*, *P. ruficapilla* and *P. stellata* across a region of southeast South Africa: (a) ratios between *F*_ST_ and *D*_PS_ genetic distance metrics within each species among sampled forest sites; (b) regional CN_e_ size of each species measured at 1% and 2% critical allele frequencies, and assuming monogomous mating (with 95% confidence intervals); (c) VarEff plots showing variation in CN_e_ of each species over the past 100 generations, assuming a single‐step mutation model at a constant mutation rate of μ = 5 × 10^−4^ per generation. Species demographic trends are inferred from a combination of eight species‐specific loci unique to each species

Amplification errors (large allele dropout, stuttering and null alleles) were checked in MICROCHECKER 2.2.3 (Van Oosterhout et al., 2006) and FREENA (Kawashima et al., 2009). Forest‐level deviations from expectations of Hardy–Weinberg equilibrium (HWE) and linkage disequilibrium (LD) within forests were assessed in GENEPOP4.7 (Rousset, 2008); adjusting significance values using a Benjamini–Hochberg correction (Benjamini & Hochberg, 1995) to control for false discovery rate. Forest‐level species genetic diversity was estimated as rarefied allelic richness (AR), and private allelic richness (PrAR) in ADZE1.0 (Szpiech et al., 2008); observed (*H*_o_) and expected (*H*_e_) heterozygosity; and inbreeding coefficient (*F*_IS_), in GENETIX4.05 (Belkhir et al., 2001). As a precautionary measure against low sample sizes and the limited number of microsatellite loci employed in this study, we used POWSIM 4.1 (Ryman & Palm, 2006) to assess the power of each microsatellite data set to detect population substructures at *F*_ST_ = 0.05 (effective population size, *N*_e_ = 2000; generations of genetic drift, *t* = 210), *F*_ST_ = 0.01 (*N*_e_ = 2000, *t* = 40), and *F*_ST_ = 0.001 (*N*_e_ = 2000, *t* = 4). An *N*_e_ of 2000, approaching the upper bounds of the estimated effective population size (see Figure 2; Table S2), was selected as larger *N*_e_ are considered more appropriate; the value of *t* was selected following recommendations by Ryman and Palm (2006) to test for the particular *F*_ST_. Simulations were performed assuming two subpopulations (*N* = 50 and *N* = 40) over 1000 replicates, and statistical power was measured as the proportion of significant tests.

Population genetic substructures were investigated through Bayesian clustering using STRUCTURE (Pritchard et al., 2000). The optimal number of genetic clusters per species (*K*) were tested for *K* = 1–12 (the number of forest sites +1). Twenty independent runs of 5 × 10^5^ Markov chain Monte Carlo (MCMC) iterations and a burn‐in period of 5 × 10^4^ were performed per *K*, using the admixture model, correlated allele frequencies, and with LOCPRIOR (grouped by forest). Results of runs averaged in STRUCTURE HARVESTER (Earl & vonHoldt, 2012), and the optimal number of clusters was determined using the Evanno Δ*K* statistic derived from posterior probability of each value of *K* (Evanno et al., 2005). STRUCTURE results were visualized in the Pophelper R package (Francis, 2017). To further investigate population structure, we performed principal component analysis (PCA) in the adegenet R package (Jombart, 2008) based on individual allele frequencies. Finally, to determine population differentiation, we calculated global and pairwise *F*_ST_ among forests for each species in ARLEQUIN 3.5 (Excoffier & Lischer, 2010).

### 2.4 Demographic history

To infer contemporary gene flow disruption, we compared pairwise *F*_ST_, estimated in ARLEQUIN 3.5, to the proportions of shared allele statistic *D*_PS_, calculated in MSA 4.0 (Dieringer & Schlötterer, 2003). Lag time to detection of new gene flow barriers is shorter when measuring *D*_PS_ compared to *F*_ST_ (Landguth et al., 2010; Robin et al., 2015; Savary et al., 2021), and so larger *D*_PS_:*F*_ST_ ratios may suggest recent reductions in gene flow (Robin et al., 2015). We estimated regional contemporary effective population sizes (CN_e_) for each species, using the LD model (for single sampling events) in NEESTIMATOR 2.1 (Do et al., 2014). We separately assumed random and monogamous mating (typically observed; Hockey et al., 2005). We observed results at 0.02 and 0.01 critical allele frequencies to better accommodate limited data sets (Do et al., 2014) and used a pairwise jackknife approach to assess confidence intervals; within‐species samples were pooled to accommodate overlap/interbreeding among the most recent generations. We further inferred variation in focal species *N*_e_ over the most recent 100 generations using the VarEff R package (Nikolic & Chevalet, 2014). Default parameter conditions were kept across species, adjusting maximum distance between alleles (DMAX = 18 – *P. stellata*; 17 – *C. dichroa*; 22 – *B. capensis*; 10 – *P. ruficapilla)*, number of past *N*_e_ changes (JMAX = 3); and generations since the most recent common ancestor (GBAR = 1000; reduced from the default GBAR = 5000 given the low population differentiation observed for each species [Nikolic & Chevalet, 2014]). Runs were performed under both single‐step mutation model (SMM), and 10% single‐step two‐phase mutation (TPM) to accommodate a broader range of mutation dynamics within natural populations. Mutation models assumed a mutation rate of μ = 5 × 10^−4^ per generation (Brohede et al., 2002; Coetzer et al., 2020), with an acceptance ratio of 0.25.

### 2.5 Landscape resistance modelling

#### 2.5.1 Landscape genetics framework

Landscape genetics frameworks provide a means to investigate relationships between genetic distances and features landscape, by modelling resistance surfaces of spatially arranged cost values to gene flow (Manel & Holderegger, 2013; Manel et al., 2003; Waits et al., 2015). To investigate the regional landscape influences on the interforest connectivity within each species, we adopted an a priori approach of resistance surface parameterization using RESISTANCEGA 4.1 R package (Peterman, 2018; Peterman et al., 2014). This approach circumvents subjectivity of conflicting expert opinion (Charney, 2012; Zeller et al., 2012) and limited applicability of niche‐model derivations towards atypical landscape use (Balkenhol et al., 2015; Keeley et al., 2017; Vasudev et al., 2015; Zhan et al., 2017). The RESISTANCEGA 4.1 R package integrates mixed‐effects modelling and stochastic genetic algorithms mimicking natural selection (Scrucca, 2013) specifically to maximize the relationship between pairwise genetic distances of samples and resistance surfaces. Models were fitted using maximum‐likelihood population effects (MLPE) parameterization (Clarke et al., 2002) in the LME4 R package (Bates et al., 2014) where fitness was assessed using corrected Akaike information criteria (AIC_c_). Models with an AICc difference (ΔAIC_c_) <2 were considered equivalent (Burnham & Anderson, 2004). We modelled two ecological distances for each landscape surface: isolation‐by‐resistance (IBR) considers cumulative current‐flow costs across all possible paths between two points (McRae, 2006), whereas least‐cost pathways (LCP) are spatial entities along which the accumulated costs are minimized (Adriaensen et al., 2003; Marrotte & Bowman, 2017). The IBR models were constructed using commute‐time resistance distances, an equivalent to circuit theory models for determining flow resistance (Lundgren & Ralph, 2019; Marrotte & Bowman, 2017; McRae et al., 2013), in RESISTANCEGA4.1. The LCP models in turn were made using the ‘cost distance’ function in the gdistance R package (Van Etten, 2015). We separately considered pairwise *F*_ST_ and pairwise *D*_PS_ (Tables S1.1–S1.4) as the dependent variable for mixed‐effects modelling, and scaled and centred LCP and IBR surfaces as predictor variables.

#### 2.5.2 Landscape variables

**FIGURE 3:**
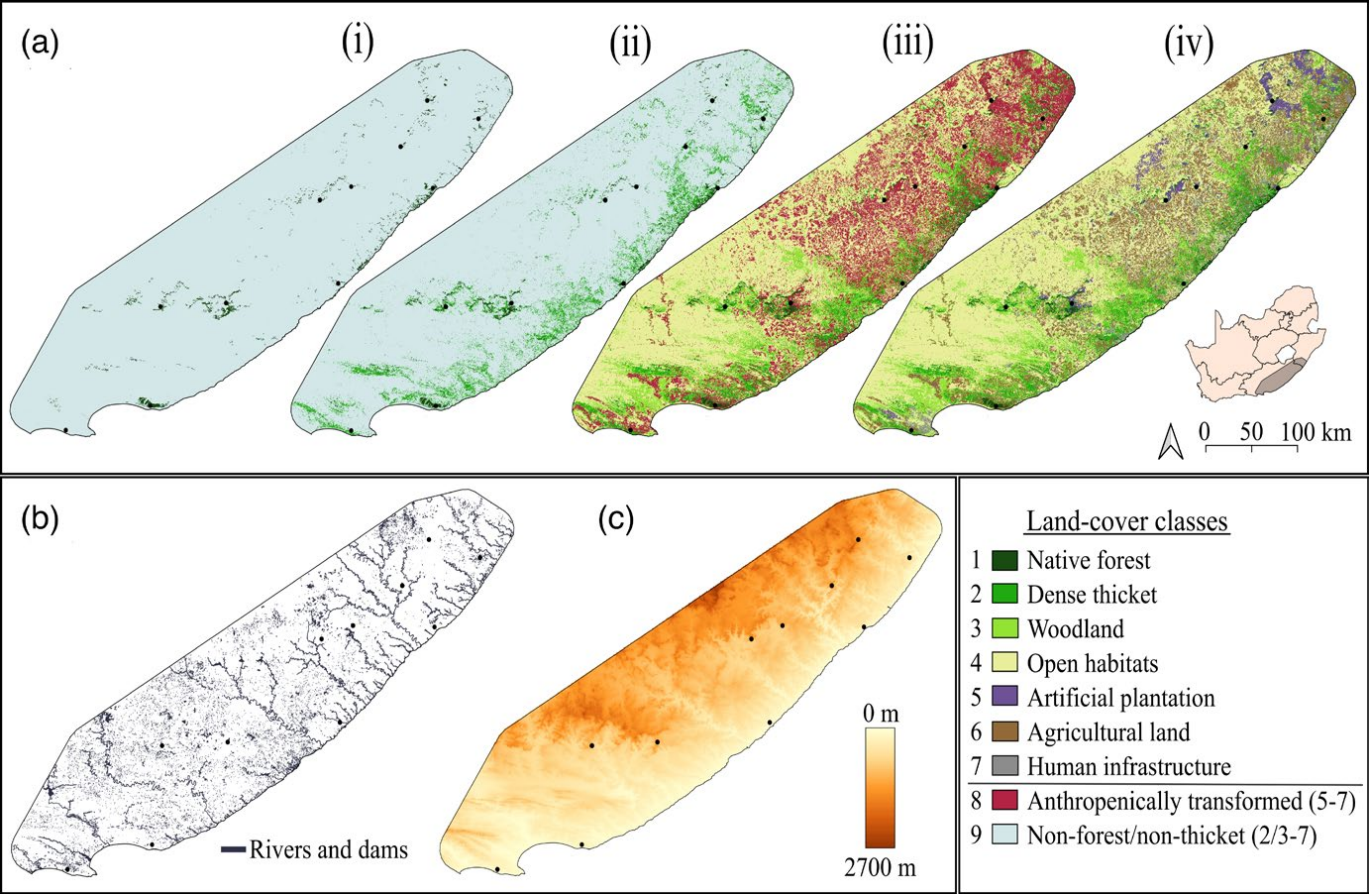
Landscape surfaces at a spatial resolution of 250 × 250 m cell size used to calculate LCP and IBR ecological distance modelling. (a) Four classifications of land‐cover surfaces: (i) native forest configuration; (ii) forest and dense thicket configuration; (iii) natural land cover (native forest, dense thicket, woodland and open habitats) and combined anthropogenically transformed land cover (artificial plantation, agricultural land and human infrastructure); (iv) all seven land‐cover classes; (b) rivers and dams; (c) elevation (in metres above sea level)

We assessed the relative influence of three landscape variables on connectivity in each focal species (Figure 3): (i) land cover, as matrix landscape has been shown to affect forest‐dependent insectivorous birds; (ii) rivers and dams, as these species may use riparian corridors both for seasonal migration, and dispersal; and (iii) elevation, as species populations may be isolated along an elevation gradient. Land cover and rivers and dams resistance surfaces were based on 20 m categorical land‐cover classes taken from the South African National Land‐Cover (SANLC) 2018 (Thompson, 2019), while topographic surfaces were based on 7.5 arc‐second (250 m) categorical map of mean elevation in metres above sea level (m.a.s.l), taken from the Global Multi‐resolution Terrain Elevation Data 2010 (Danielson & Gesch, 2011). We down‐scaled the resolution of all resistance surfaces to 250 m × 250 m for computational efficiency without significant loss of landscape‐genetic associations (McRae et al., 2008). For riparian corridors, we grouped rivers, estuaries, dams, and herbaceous wetland elements and classified every cell containing these elements as ‘river’; we then added a single‐cell buffer around all ‘rivers’ to prevent diagonal side‐stepping when modelling LCP and IBR under an 8‐connexity parameter. For land cover (see below), we classified cells according to the majority land‐cover class within each cell. We prepared resistance surfaces by creating a convex hull around forest sites using the Convex Hull plug‐in for QGIS3.10 (QGIS Development Team, 2019), and setting a 50‐km buffer as the boundary for downstream analyses. The latter condition was included to accommodate total movement among available forest habitat throughout the study area.

#### 2.5.3 Thematic resolution of landscape matrix

**FIGURE 4:**
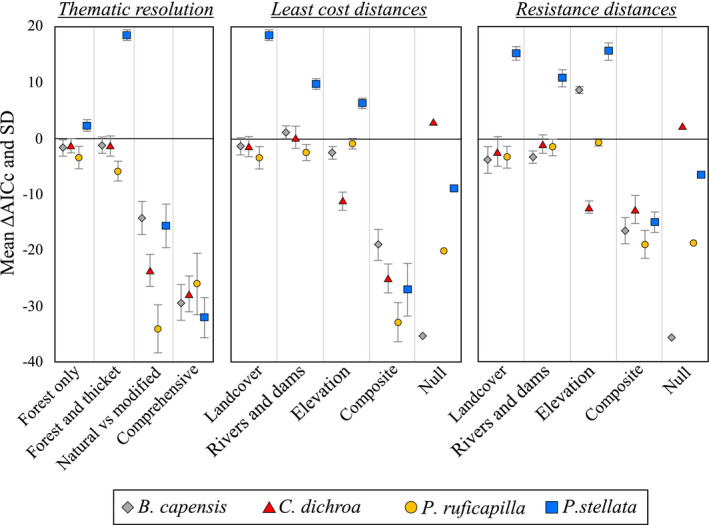
Relative performance of least‐cost pathway and resistance distance models based on landscape surfaces for the four focal bird species, inferred from *F*_ST_. Univariate optimizations were conducted independently on four land‐cover thematic surfaces, modelling least‐cost paths (left). Univariate optimizations were also conducted separately for best‐supported land‐cover, rivers and dams, and elevation; and multivariate optimizations integrated the three landscape layers into a composite surface. Both univariate and multivariate optimizations employed three replicates of least‐cost (middle), and resistance distance (right) modelling regimes. Positive ΔAICc values denote improved model performance over Euclidean distances

To infer matrix permeability across different land‐cover classes, we tested four alternative land‐cover surfaces under different classification schemes (Figure 3a). The original 72 land‐cover classes were consolidated into seven categories: (a) native forest (≥75% tree canopy cover; ≥6 m canopy height); (b) dense thicket—coastal/mesic/valley thicket (≥75% tree canopy cover; 2.5–6 m canopy height); (c) woodland (35–75% tree canopy cover; ≥2.5 m canopy height); (d) open habitats—grassland, shrubland and savanna (<35% tree canopy cover); (e) artificial plantation (exotic *Pinus*/*Eucalyptus*); (f) agriculture; and (g) human infrastructure (urban, suburban, rural, industrial, transportation networks and mining). From this, four resistance surfaces were classified as follows: (1) native forest configuration (the primary habitat of each species); (2) forest and dense thicket configuration, as the latter habitat improves forest bird community connectivity; (3) natural land cover (forest, dense thicket, woodland, open habitats) versus anthropogenically transformed land cover (exotic plantation, agricultural land, human infrastructure); and (4) a comprehensive surface comprising all seven categories. For computational efficiency, land‐cover resistances surfaces 1–4 were optimized three times per species, using least‐cost distances based on pairwise *F*_ST_. Given that *D*_PS_ better responds to recent landscape change landscape change (Savary et al., 2021), we optimized the these resistance surfaces once using this genetic distance metric, as *F*_ST_ results proved stable across replicates (Figure 4). The best‐supported thematic resolution for each species was used in subsequent analyses.

#### 2.5.4 Resistance surface optimizations and landscape distance model comparisons

Univariate optimization was conducted in RESISTANCEGA 4.1, separately on the three landscape resistance surfaces (land cover, riparian corridors and elevation), using both LCP and IBR modelling. Subsequently, we conducted multivariate optimization, wherein the optimizations of all three surfaces are summed to form a composite surface over which LCP and IBR distances are modelled. All optimizations (except previously optimized land‐cover LCP models) were optimized three times, separately for each species. All IBR and LCP models were compared to null models. Following Cushman et al. (2013) and Khimoun et al. (2017), we used causal modelling to compare species‐specific IBR and LCP ecological distance models to isolation‐by‐distance (IBD), according to the Spearman correlations between genetic and landscape distances. Despite the high rates of type I error alleged for partial Mantel tests (Castellano & Balletto, 2002; Raufauste & Rousset, 2001), use of these tests in a causal model framework to reject the incorrect causal model and to identify the most applicable models driving observed genetic patterns is considered appropriate (Cushman & Landguth, 2010; Cushman et al., 2013; Khimoun et al., 2017). We inferred the most relevant landscape model using Mantel and partial Mantel tests in the ecodist R package (Goslee & Urban, 2015), with 10,000 random permutations, using distance values for LCP and IBR models, and log‐transformed Euclidean distances for IBD. Spatially constrained, yet otherwise indiscriminate dispersal should best reflect IBD model. Dispersal which navigates select routes across landscape elements efficiently should reveal LCP, whereas inefficient dispersal, or dispersal across numerous paths through landscape elements should show IBR.

## 3 RESULTS

### 3.1 Microsatellite characteristics and genetic diversity

All individuals were successfully genotyped for all loci (Table 1). The eight informative microsatellite markers retained per species exhibited no large allele dropout, or stuttering, displayed null allele frequencies <5% across populations, and had limited deviations from expectations of LD and HWE (Appendix S1–S3). At the forest level, loci were in HWE after Benjamini–Hochberg corrections, except two loci in *B. capensis* (BMI‐71 at Nqadu, and BMI‐98 at The Island). Only 2/252 (0.79%) LD tests concerning different loci/population combinations in *P. stellata* remained significant after Benjamini–Hochberg corrections, while no significant linkage was evident within the other species‐specific sets of loci (in *P. ruficapilla*, a ninth locus, POCC8 was discarded for displaying null alleles, and significant linkage to POCC9 across all sites [Appendix S1]). All loci retained were therefore assumed independent. Genetic diversity, in terms of standardized allelic richness and observed heterozygosity, was similar between *B. capensis* (AR = 10.625 ± 1.102; *H*_o_ = 0.625 ± 0.232), *P. stellata* (AR = 9.750 ± 0.794; *H*_o_ = 0.646 ± 0.186), and *C. dichroa* (AR = 8.00 ± 0.845; *H*_o_ = 0.648 ± 0.152), yet lowest in *P. ruficapilla* (AR = 3.375 ± 0.443; *H*_o_ = 0.391 ± 0.102) (Table 1). Private allelic diversity was highest in *C. dichroa* (PrAR = 0.264 ± 0.089), slightly lower in *B. capensis* (PrAR = 0.196 ± 0.082) and *P. stellata* (PrAR = 0.185 ± 0.067), and very low in *P. ruficapilla* (PrAR = 0.043 ± 0.031) (Table 1). *Batis capensis* exhibited the highest inbreeding coefficient (overall *F*_IS_ = 0.151) across most forest sites, whereas *F*_IS_ estimates in the other three species were close to zero (Table 1; Appendix 2). Microsatellite performance assessments showed it was possible to detect genetic divergence as low as *F*_ST_ = 0.01 (*t* = 40) with 94.4% certainty in *B. capensis*, 92.4% in *C. dichroa* and 91.1% in *P. stellata*, but only 55.7% certainty for *P. ruficapilla*, although *F*_ST_ = 0.02 (*t* = 80, *n* = 2000) could be detected with 84.7% certainty for this species. All four microsatellite data sets had low certainties (6.5%−12.0%) to detect *F*_ST_ = 0.001 (*t* = 4).

### 3.2 Genetic population structure

**FIGURE 5:**
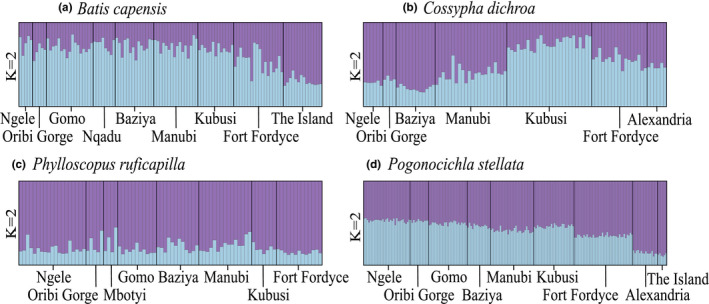
STRUCTURE assignment plots for *K* = 2 for (a) *B. capensis*, (b) *C. dichroa*, (c) *P. ruficapilla* and (d) *P. stellata*. Each line represents the admixture proportions within one individual, and individuals were grouped according to sampled forest sites across southeast South Africa. Admixture proportions within each species were respectively inferred from a unique combination of eight species‐specific microsatellite loci

**FIGURE 6:**
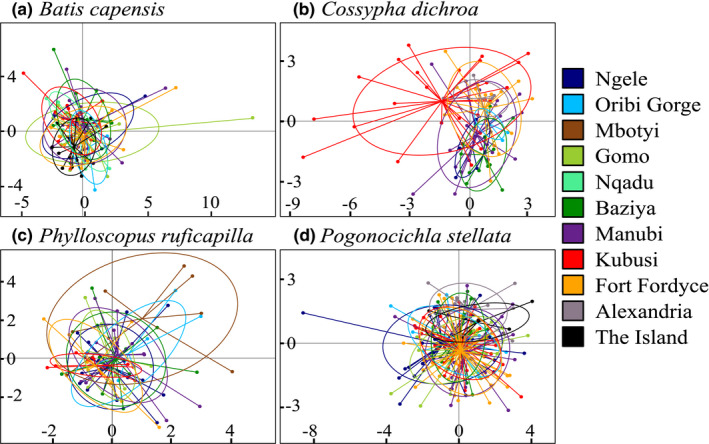
Principal component analysis plots for (a) *B. capensis*, (b) *C. dichroa*, (c) *P. ruficapilla* and (d) *P. stellata*, respectively, based on individual allele frequencies from eight species‐specific microsatellite loci

Global genetic differentiation was significant for *C. dichroa* (*F*_ST_ = 0.036, *p* < 0.001) and *P. stellata* (*F*_ST_ = 0.016, *p* < 0.001), but not for *B. capensis* (*F*_ST_ = 0.013, *p* = 0.100) and *P. ruficapilla* (*F*_ST_ = 0.006, *p* = 0.261). STRUCTURE identified a maximum of two genetic clusters within the regional populations of each species, respectively, according to Evanno Δ*K*: *B. capensis* (Δ*K* = 7.275), *C. dichroa* (Δ*K* = 1.184), *P. ruficapilla* (Δ*K* = 1.099) and *P. stellata* (Δ*K* = 3.034) (Figure 5a–d, Figure S1.1–S1.4). In both *B. capensis* and *P. stellata*, there is a subtle north–south gradient, wherein individuals from the southern IOCB forests (Alexandria and The Island) appear partially differentiated from individuals within Ngele to Fort Fordyce (Figure 5a,d). This gradient is less apparent in *P. stellata* where individuals from Ngele to Fordyce showed higher mixed ancestry compared to *B. capensis* (Figure 5a,d). In *C. dichroa*, there is also a subtle north–south gradient, although individuals from Kubusi appear particularly differentiated from other regional forests (Figure 5b). Finally, in *P. ruficapilla*, the population structure appears uniform across the study area (Figure 5c). The PCA results suggested that all four species, individuals across regional forests broads comprise genetically homogenous groups (Figure 6a–d). However, in *C. dichroa*, individuals from Kubusi show large variability and partially cluster away from other forest groups (Figure 6b), and this is similarly observed in *P. ruficapilla* for individuals from Mbotyi (Figure 6c).

### 3.3 Demographic history

Pairwise *D*_PS_:*F*_ST_ ratios were highest for *B. capensis*, *P. stellata* and *C. dichroa*, and lowest for *P. ruficapilla* (Figure 2a). Overall CN_e_ appears lowest in *C. dichroa* and highest in *P. ruficapilla*, although both have larger 95% *CI* compared to *P. ruficapilla* and *P. stellata* (Figure 2b; Table S2). Species CN_e_ assuming monogamous mating (typically observed in each species [Hockey et al., 2005]) were twice as high compared to assuming random mating (Table S2). Disparities between CN_e_ at 1% and 2% critical allele frequencies were minimal in *B. capensis*, 18% in *P. stellata*, 33% in *C. dichroa* and 150% for *P. ruficapilla*, reflecting lower rare allele frequencies in the last three species (Do et al., 2014). Fluctuations in *N*_e_ over the past 100 generations varied across the four songbirds, consistent across single‐step (Figure 2c), and two‐phase (Figure S2) mutation models. Historically, *B. capensis* and *C. dichroa* had the largest *N*_e_, but declined to levels comparable to *P. ruficapilla* and *P. stellata*, which both appear more temporally stable, but still in decline (Figure 2c). Assuming a two‐year generation time (Bird et al., 2020), or three years for *P. stellata* (Oatley, 1982b), these events relate to the past three centuries, with most declines beginning <100 years (~20–60 generations) ago.

### 3.4 Landscape genetics

#### 3.4.1 Land‐cover thematic resolution

Land‐cover thematic surface evaluation (Figure 4) indicated that genetic distances in *P. stellata* were best explained by the native forest and dense thicket configuration model, which outranked the geographic distance model by >17 AICc units. For *B. capensis* and *C. dichroa*, both the native forest configuration, and the native forest and dense thicket configuration model were comparable to the geographic distance model (<2 AICc units) (Figure 4). However, in *P. ruficapilla*, the native forest configuration model ranked second to the geographic distance model (<4 AICc units) (Figure 4). For each species, land‐cover models ranked similarly across pairwise *F*_ST_ and *D*_PS_ (Figure 4, Figure S3), except for *C. dichroa*, in which the null model outranked all other models when using pairwise *F*_ST_ (Figure 4), but ranked below the geographic distance model, and native forest and dense thicket configuration model by<4 AICc units (Figure S3). According to both pairwise *F*_ST_ and *D*_PS_, detailed land‐cover configurations were inadequate to explain genetic distances observed in the four species (Figure 4; Figure S3).

#### 3.4.2 Landscape resistance surfaces

For *B. capensis*, only the resistance distance model from elevation outranked the geographic distance model (>8 AICc units), although least‐cost distance models from both land‐cover (native forest and dense thicket configuration), and rivers and dams were comparable to the geographic distance model (Figure 4). For *C. dichroa*, the null model outranked all landscape models (Figure 4). For *P. ruficapilla*, the least‐cost distance model from elevation, and the resistance distance models from elevation, and rivers and dams were comparable to the geographic model (Figure 4). Only in *P. stellata* did univariate least‐cost and resistance distance models from each univariate landscape surface consistently outranked the geographic distance model (Figure 4), of which the least‐cost distance model from land cover (native forest and dense thicket configuration) ranked the highest (>17 AICc). Across all four species, the least‐cost and resistance distance models from the composite landscape surface ranked far lower than the geographic distance model (Figure 4).

#### 3.4.3 Comparative performance of landscape models

**FIGURE 7:**
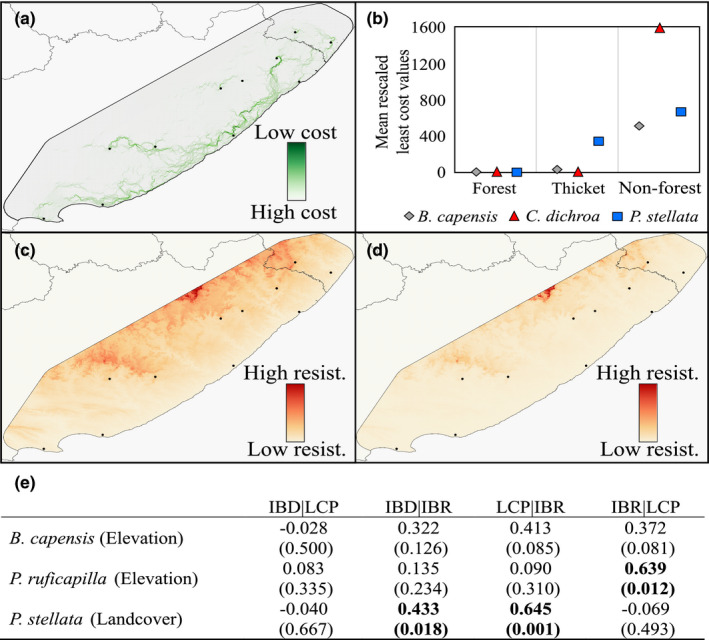
Most relevant optimized landscape surfaces impacting regional gene flow within the four forest‐dependent birds. (a) Current flow density representing least‐cost pathways through native forest and dense thicket land cover—the most relevant landscape resistance model in *P. stellata*; (b) mean rescaled least‐cost values for native forest, dense thicket and nonforest land‐cover classes for *B. capensis*, *C. dichroa* and *P. stellata*, respectively; (c) optimized elevation resistance surface for *B. capensis*, and for (d) *P. ruficapilla*; causal modelling between IBD and LCP and IBR models of the most relevant landscape resistance surfaces for *B. capensis*, *P. ruficapilla* and *P. stellata*

Both *B. capensis* add *P. stellata* showed significant IBD according to pairwise *F*_ST_ (Table 2), whereas *C. dichroa* and *P. stellata* showed significant IBD according to pairwise *D*_PS_ (Table 3). Partial Mantel tests of either LCP or IBR models controlling for IBD suggest that for *B. capensis*, *P. ruficapilla* and *P. stellata*, genetic distances (pairwise *F*_ST_) better correlated with certain landscape elements than geographic distance (Table 2). In *B. capensis*, genetic distances correlated significantly with the IBD‐controlled LCP (LPC|IBD) models for native forest and dense thicket, rivers and dames, and landscape elevation (Table 2). In this species, landscape elevation appears especially pertinent to gene flow, with both LCP|IBD and IBR|IBD models for elevation strongly correlating to genetic distances (Table 2). Causal modelling, however, did not support one ecological distance model of the other (Figure 7). In *P. ruficapilla*, genetic distances correlated strongly with IBR|IBD models with native forest land‐cover, rivers and dams, and especially with landscape elevation (Table 2). In this species, causal modelling showed that the IBR model of landscape elevation remained significant even after controlling for LCP (Figure 7). In *P. stellata*, genetic distances correlated with all tested ecological distance models (after controlling for IBD), but especially so for the LCP|IBD model for native forest and dense thicker land cover (Table 2). The association between genetic distances within *P. stellata* and these land‐cover classes remained significant across both pairwise *F*_ST_ (Table 2), and pairwise *D*_PS_ (Table 3). In this species, causal modelling further corroborated the LCP model over the IBR model for these land‐cover classes, further showing that IBD may better explain genetic distances than this land‐cover IBR model (Figure 5). Although no landscape model pairwise *F*_ST_ genetic distances in *C. dichroa* (Table 2), the LCP model for both native forest, and native forest and dense thicket remained significant after controlling for IBD (Table 3).

**TABLE 2:** Partial Mantel tests comparing Spearman's correlations between landscape features and genetic distances (*F*_ST_) for four forest‐dependent songbird species across a region of southeast South Africa. Least‐cost pathways (LCP) and isolation‐by‐resistance (IBR) ecological distances modelled for resistance surface controlled for isolation‐by‐distance (IBD) (shown in the bottom row). Only landscape resistance surfaces which ranked above or equal to the geographic distance model (Figure 4) are included. Bold indicates significantly supported correlations (*p* < 0.05) (shown in parentheses)

Landscape surface	*B. capensis*		*C. dichroa*		*P. ruficapilla*		*P. stellata*	
	LCP|IBD	IBR|IBD	LCP|IBD	IBR|IBD	LCP|IBD	IBR|IBD	LCP|IBD	IBR|IBD
Native forest	0.345 (0.119)	0.247 (0.306)	0.147 (0.217)	0.335 (0.071)	0.290 (0.122)	**0.367 (0.038)**	**0.550 (0.007)**	**0.403 (0.0351)**
Native forest and dense thicket	**0.509 (0.006)**	0.129 (0.300)	0.269 (0.229)	0.227 (0.278)	0.335 (0.070)	0.218 (0.334)	**0.772 (0.002)**	**0.562 (0.024)**
Rivers and dams	**0.447 (0.031)**	0.351 (0.102)	0.247 (0.140)	0.206 (0.334)	0.286 (0.092)	**0.309 (0.031)**	**0.599 (0.014)**	**0.387 (0.028)**
Elevation	**0.855 (0.001)**	**0.850 (0.001)**	0.371 (0.175)	0.395 (0.165)	0.527 (0.070)	**0.752 (0.003)**	**0.698 (0.014)**	**0.657 (0.015)**
Geographic distance (IBD)	**0.404 (0.048)**		0.281 (0.109)		0.338 (0.061)		**0.530 (0.003)**	

**TABLE 3:** Partial Mantel tests (controlling for isolation‐by‐distance) comparing Spearman's correlations between landscape and genetic distances (*D*_PS_) for focal species populations across a region of southeast South Africa. Only least‐cost pathways (LCP) are modelled for each of the two land‐cover resistance surfaces which ranked either above or equal to the geographic distance model of Figure S3. Bold indicates significantly supported correlations (*p* < 0.05) (shown in parentheses)

Landscape surface	*B. capensis*	*C. dichroa*	*P. ruficapilla*	*P. stellata*
Native forest	0.015 (0.392)	**0.422 (0.022)**	−0.090 (0.585)	0.206 (0.194)
Native forest and dense thicket	0.207 (0.199)	**0.445 (0.025)**	−0.200 (0.703)	**0.353 (0.022)**
Geographic distance (IBD)	0.073 (0.312)	**0.420 (0.013)**	−0.123 (0.670)	**0.349 (0.013)**

## 4 DISCUSSION

The four forest‐dependent insectivorous songbirds in this study adequately maintain gene flow among spatially isolated metapopulations confined to the fragmented native forests of southeast South Africa (Figure 5). Gene flow disruptions are however notable in *B. capensis*, and to a lesser extent in *P. stellata* and *C. dichroa* (Figure 2a). Two species, *B. capensis* and *C. dichroa*, show pronounced declines in effective population sizes (Figure 2c), signifying vulnerability of these avian insectivores to increasing anthropogenic habitat changes. The endemic forest‐specialist *C. dichroa* has experienced the most drastic population declines over the past two centuries, which have continued well after the widespread cessation of commercial selective logging and deforestation 80 years ago.

### 4.1 Population genetic structures

The South African endemic *C. dichroa* displayed the highest population structuring (Figure 5a), and the large genetic variability unique to Kubusi (Figure 6b) affirms the climatic refugial importance of the eastern Amatole forest complex (Dalton et al., 2015; Kushata et al., 2020; Madisha et al., 2018). The higher population complexity of this forest specialist contrasts with that of the forest generalist *B. capensis*, which is near endemic to South Africa, and more genetically diverse than *C. dichroa* (Table 1). Higher historic availability of suitable habitat for *B. capensis* likely afforded larger populations that were more buffered against Paleoclimatic fluctuations, although both native forest (Ivory et al., 2018; Lawes, Eeley, et al., 2007) and Albany thicket (Potts et al., 2013) biomes were susceptible to contractions during periodic aridity. Consistently higher genetic diversity at Manubi across species (Table 1) corroborates the refugial significance of intermediate (scarp) forests within South Africa (Grass et al., 2015; Lawes, Eeley, et al., 2007; Moir et al., 2020; Moir et al., 2021), which are close to the coast, buffering them from palaeoclimatic extremes. The lower diversity at Oribi Gorge may reflect greater proximity to subtropical IOCB forests (Mucina, 2018; Mucina et al., 2006), which re‐established only ~8 kya (Huntley et al., 2016) and are generally avoided by these four songbird species. The low genetic diversity observed in *P. ruficapilla* suggests more recent, or perhaps constrained, colonization of South Africa than does *P. stellata*, and the unexpectedly low regional complexity within *P. ruficapilla* contrasts with the strong population insularity observed in East Africa (Callens et al., 2011). This alludes to dispersal behaviour of this species changing from sedentary in tropical Africa, to vagile in higher latitude forests of South Africa (Martin & Tewksbury, 2008; Moore et al., 2008; Salisbury et al., 2012).

### 4.2 Population viability and effective population size

Our study findings suggest that historic deforestation and commercial selective logging likely had a large negative impact on the viability of these four species (Figure 2c), more so than contemporary informal forest resource harvesting, despite it being largely unregulated (Leaver & Cherry, 2020a, 2020b; Leaver et al., 2019). The contemporary effective population sizes of all four species (Figure 2b; Table S2) are likely underestimates resulting from pooled generations (Luikart et al., 2010), although true numbers are likely to remain low. Surprisingly, *B. capensis* exhibited the most restricted gene flow and highest signs of inbreeding (Table 1), despite showing the lowest range contraction (−1.3%), and broadest habitat tolerance, extending into dense thicket. The Albany thicket biome has experienced minimal loss (−8.9%) between 1750 and 2014 (Skowno et al., 2019), and total thicket vegetation has steadily increased across the Eastern Cape since 1950 (Njwaxu & Shackleton, 2019; Skowno et al., 2019; Stickler & Shackleton, 2014). But 63% of Albany thicket is severely degraded (14.0–25.4% valley thicket, and 12.8% mesic thicket) (Lloyd et al., 2002), and the extent of coastal thicket degradation is unknown. The cumulative effect of both native forest and dense thicket transformation has likely affected *B. capensis* more adversely than either *P. stellata* or *C. dichroa*, which probably use dense thicket only for facultative dispersal. Recuperation of the Albany thicket sub‐biome augurs well for the population recovery of *B. capensis*, due to greater habitat availability, as well as the improved viability of *P. stellata* and *C. dichroa*. Improved landscape resistance modelling performance according to pairwise *D*_PS_ for *C. dichroa* (Table 3) may reflect species recovery.

In east Africa, post‐fragmentation sensitivity is evident for *P. ruficapilla* and *P. stellata* (Callens et al., 2011; Githiru & Lens, 2006; Sirén et al., 2018), yet the populations of both species in the Eastern Cape appear to have been largely resilient to historic forest exploitation. Afrotropical forest‐dependent species have been observed to initially display stable effective population sizes following forest fragmentation (Husemann et al., 2015; Lens et al., 2002), but continued forest degradation eventually undermines population viability (Habel et al., 2014; Korfanta et al., 2012; Lens & Van Dongen, 1999). In South Africa, this is observable in both *P. ruficapilla* and *P. stellata* (Figure 2c).

### 4.3 Gene flow among regional forests

Metapopulation dynamics of these four songbird species do not appear to wholly contingent upon observed adult mobility. Observed regional adult vagility in *B. capensis* and *P. stellata* (Oschadleus & Ranwashe, 2017) did not preclude long‐term (*F*_ST_) isolation‐by‐distance in these species (Table 2). Local seasonal migration observed elsewhere in *C. dichroa* (Johnson & Maclean, 1994; Oatley et al., 2017; Oatley, 1966, 1969) is contested within southeast South Africa (Craig & Hulley, 2019; Wolmarans, 2015). Stronger population differentiation (Figure 5) and more recent (*D*_PS_) regional isolation‐by‐distance in this species indicates that regional populations of this species are likely sedentary. (Table 3). The apparent panmixia (Figure 5) and lack of isolation‐by‐distance (Table 2) observed within *P. ruficapilla* strongly indicates underestimated regional dispersal ability of this species.

However, philopatry—the tendency for vagile organisms to return to the same breeding location—has been observed in *P. stellata* (Dowsett, 1985; Oatley, 1982a) and *C. dichroa* (Oatley, 2017); strong adult site fidelity is observed in all four species (Callens et al., 2011; Habel et al., 2016; Oatley, 1982a; Oschadleus & Ranwashe, 2017) and attested to by recapture records (Oschadleus & Ranwashe, 2017). Adult philopatry (Habel et al., 2016; Oatley, 1982a) could mean that gene flow is mostly contingent upon natal dispersal of young birds (Garrard et al., 2012). Natal dispersal of birds is poorly documented in South Africa, but has been observed in *P. stellata* (Oatley, 1982a), with 2‐ to 3‐month‐old individuals having been observed moving through plantations, woodland and riparian thicket. Intuitively, young *P. stellata* should seek to minimize exposure and attempt cost‐efficient navigation of hospitable matrix vegetation, explaining the high performance of the LCP model of forest and coastal/mesic thicket configuration.

### 4.4 Species–landscape interactions

Landscape resistance modelling suggests that the configuration of both native forest, and dense (coastal/mesic/valley) thicket is important to gene flow in *B. capensis*, *C. dichroa* and *P. stellata* (Figures 4 and 7; Tables 2 and 3). *Pogonocichla stellata* demonstrates higher gene flow resistance through these thicket habitats compared to *B. capensis* and *C. dichroa* (Figure 7), potentially indicating that *P. stellata* disperses facultatively through select thicket habitats, while *B. capensis* and *C. dichroa* are more inclined to inhabit these habitats (especially mesic Albany thicket adjacent to native forests) in southeast South Africa (Johnson, 1997; Oatley, 1997a). Dispersing *P. ruficapilla* appear not to discriminate land‐cover beyond forest configuration (Figure 4). This weak land‐cover association could be due to a type I error derived from low sample size (Winiarski et al., 2020), but the near panmixia within *P. ruficapilla* (Figure 5), and equilibrium between historic and contemporary gene flow (Figure 2b) supports the notion of high dispersal within this species, and tolerance towards anthropogenic landscape transformation.

The significant influence of regional elevation on the population structure of *B. capensis*, *P. ruficapilla* and *P. stellata* (Figure 7; Table 2) could indicate an elevation gradient to gene flow, potentially supporting altitudinal migration in *P. stellata*. For these three species, lower elevations appear more conducive to dispersal (Figure 7), with ravines, valleys and gorges serving as conduits into interior Afromontane forests. Outperformance of IBR over LCP elevation models respectively demonstrate inefficient navigation of landscape elevation, or interference by other landscape features. Rivers and dams appear to impact the gene flow of *B. capensis*, *P. ruficapilla* and *P. stellata* (Table 2). This landscape genetic association is similarly observed in a forest‐associated pipistrelle (Moir et al., 2020).

### 4.5 Implications for regional Afromontane forest bird conservation

Stronger performance of the LCP models of native forest and dense thicket configuration over respective IBR models for *P. stellata* (Figure 7e), as well as *B. capensis* and *C. dichroa* (Tables 2 and 3) demonstrate the potential utility of conservation corridors in the Eastern Cape and southern KwaZulu‐Natal to preserve genetic integrity within regional Afromontane forest birds. Such corridors should promote resilience anthropogenic climate change, as recommended by Colyn et al. (2020). The highest priority forests for conservation are the scarp forests present along the Wild Coast, as well the eastern Amatole Afromontane forests, and Afromontane (eastern mistbelt) forests in southern KwaZulu‐Natal. These forests harbour the largest overall and unique genetic diversity (Table 1) and are therefore the most likely to serve as future climatic refugia. Effective creation of conservation corridors could incorporate forest and coastal/mesic thicket vegetation at lower elevations, particularly where these two land‐cover classes coincide with rivers and dams, to ensure the preservation of optimal dispersal pathways beneficial for these four species. The utility of coastal thicket in facilitating movement of forest‐dependent taxa is already recognized, and many authorities regard coastal thicket as secondary forest (Ehlers‐Smith et al., 2018a, 2019; Ehlers‐Smith, Ehlers‐Smith, Ramesh, et al., 2017; Olivier et al., 2013). Beyond forest and dense thicket configuration, matrix land cover was not shown to impact the gene flow of these four songbird species significantly. Avian connectivity between IOCB forests in KwaZulu‐Natal can remain high across anthropogenically transformed areas, but minimally in forest‐dependent insectivores (Neuschulz et al., 2013). Matrix transformation, however, can diminish regional South African forest ecological integrity (Botzat et al., 2015; Ehlers‐Smith et al., 2019, 2020; Ehlers‐Smith, Si, Ehlers‐Smith, Kalle, et al., 2018; Freeman et al., 2018) and undermine the population viability of forest‐dependent birds within forests.

## 5 CONCLUSION

Our results show that reported forest‐dependent range contractions in four insectivorous birds do not closely reflect species genetic responses to anthropogenic activity within the study area of the Eastern Cape and southern KwaZulu‐Natal provinces of South Africa. The forest generalist *B. capensis* underwent the lowest range contraction (−1.3%), yet showed the most substantial gene flow restrictions, alongside pronounced declines in effective population size. More extensive range contractions observed in *P. ruficapilla* (−20.7%) and *P. stellata* (−23.0%) do not correspond to the comparatively stable effective population sizes observed, although gene flow restriction is evident in the latter species. Only the South African endemic forest specialist *C. dichroa* showed simultaneous declines in distribution (−19.5%) and effective population size, alongside gene flow disruption, and thus appears especially vulnerable to forest degradation.

In all four species, landscape resistance modelling suggested that regional gene flow within each of the four species is likely affected by landscape features. Native forest and dense thicket configuration is important to gene flow in *P. stellata*, *B. capensis* and *C. dichroa*, with *B. capensis* seeming most averse to thicket degradation. Beyond dense thicket, all four species, but particularly *P. ruficapilla*, do not facultatively disperse through matrix land cover. Finally, we propose that by conserving optimal dispersal routes through the two land‐cover classes, predominately within low‐elevation regions and coinciding with prominent river systems, should effectively ameliorate gene flow disruption and mitigate extinction debts culminating from historic forest exploitation.

## CONFLICT OF INTEREST

The authors declare no conflicts of interest.

## Supporting information

Supplementary Material

Appendix S1‐S3

## Data Availability

Data for this study will be available at the Dryad Digital Repository: to be completed after manuscript is accepted for publication. Microsatellite genotyping: https://doi.org/10.5061/dryad.rr4xgxd7b.
