# Supplementary material for: Species–landscape interactions drive divergent population trajectories in four forest‐dependent Afromontane forest songbird species within a biodiversity hotspot in South Africa
Source: Evol Appl. 2021 Oct 28;14(11):2680–97. doi: 10.1111/eva.13306 (PMC8591328; doi:10.1111/eva.13306)

**Title:** Species-landscape interactions drive divergent population trajectories in four forest-dependent Afromontane forest songbird species within a biodiversity hotspot in South Africa

Supplementary materials: Table S1.1-S1.4,

**Table S1.1** Pairwise *F*_ST_ and pairwise *D_PS_* (below and above diagonal, respectively) values for *B. capensis* (global *F_ST_* = 0.013, *p* = 0.10) based on eight microsatellite loci. * denotes significant *F_ST_* values (*p* <0.05)

|  | Ngele | Oribi Gorge | Gomo | Nqadu | Baziya | Manubi | Kubusi | Fort Fordyce | The Island |
| --- | --- | --- | --- | --- | --- | --- | --- | --- | --- |
| Ngele | - | 0.7 | 0.8 | 0.84127 | 0.78571 | 0.81122 | 0.82143 | 0.78571 | 0.82381 |
| Oribi Gorge | **0.08109*** | - | 0.45714 | 0.41429 | 0.40536 | 0.39592 | 0.36224 | 0.33846 | 0.3381 |
| Gomo | **0.04497*** | 0.02148 | - | 0.40635 | 0.38036 | 0.41531 | 0.40204 | 0.37143 | 0.41429 |
| Nqadu | **0.0693*** | 0.03944 | 0.01285 | - | 0.33829 | 0.28685 | 0.31689 | 0.31746 | 0.33175 |
| Baziya | 0.01803 | **0.04918*** | 0.016 | 0.00843 | - | 0.32781 | 0.27423 | 0.27747 | 0.2869 |
| Manubi | 0.0246 | 0.03712 | 0.01137 | 0.01464 | 0.01107 | - | 0.22959 | 0.27276 | 0.26429 |
| Kubusi | 0.0153 | 0.031 | 0.01856 | 0.0171 | 0.0111 | 0.0071 | - | 0.24137 | 0.23401 |
| Fort Fordyce | 0.0265 | 0.03101 | 0.0104 | 0.01606 | 0.00888 | 0.0257 | 0.00386 | - | 0.2652 |
| The Island | **0.0428*** | 0.03091 | 0.02007 | 0.02022 | 0.02632 | **0.02419*** | 0.0162 | 0.01997 | - |

**Table S1.2** Pairwise *F*_ST_ and pairwise *D_PS_* (below and above diagonal, respectively) values for *C. dichroa* (global *F_ST_* = 0.036, *p* < 0.001) based on eight microsatellite loci. * denotes significant *F_ST_* values (*p* <0.05)

|  | Ngele | Oribi Gorge | Baziya | Manubi | Kubusi | Fort Fordyce | Alexandria |
| --- | --- | --- | --- | --- | --- | --- | --- |
| Ngele | - | 0.51429 | 0.67857 | 0.66667 | 0.69156 | 0.68254 | 0.63265 |
| Oribi Gorge | **0.08776*** | - | 0.41071 | 0.37738 | 0.35844 | 0.43095 | 0.43469 |
| Baziya | 0.02252 | **0.11502*** | - | 0.43452 | 0.33442 | 0.4246 | 0.44643 |
| Manubi | **0.03069*** | 0.02047 | **0.01537*** | - | 0.26353 | 0.37698 | 0.37585 |
| Kubusi | **0.03966*** | **0.05405*** | **0.06054*** | **0.0158*** | - | 0.26479 | 0.36132 |
| Fort Fordyce | **0.04254*** | **0.07338*** | **0.04485*** | **0.02070*** | **0.01884*** | - | 0.39966 |
| Alexandria | **0.05904*** | **0.14447*** | **0.08728*** | **0.05107*** | **0.04691*** | **0.0589*** | - |

**Table S1.3** Pairwise *F*_ST_ and pairwise *D_PS_* (below and above diagonal, respectively) values for *P. ruficapilla* (global *F_ST_* = 0.003, *p* = 0.261) based on eight microsatellite loci. * denotes significant *F_ST_* values (*p* <0.05)

|  | Ngele | Oribi Gorge | Mbotyi | Gomo | Baziya | Manubi | Kubusi | Fort Fordyce |
| --- | --- | --- | --- | --- | --- | --- | --- | --- |
| Ngele | - | 0.5119 | 0.54286 | 0.53571 | 0.48052 | 0.5 | 0.52143 | 0.50794 |
| Oribi Gorge | 0.01864 | - | 0.12857 | 0.25 | 0.1342 | 0.17262 | 0.12381 | 0.19841 |
| Mbotyi | 0.038 | 0.00873 | - | 0.24643 | 0.15974 | 0.17738 | 0.10714 | 0.23968 |
| Gomo | 0.033 | 0.01409 | 0.02537 | - | 0.21429 | 0.2381 | 0.2 | 0.22222 |
| Baziya | 0.047 | 0.0362 | 0.0361 | 0.0493 | - | 0.14989 | 0.12857 | 0.19048 |
| Manubi | 0.0213 | 0.05115 | 0.06081 | 0.02 | 0.03162 | - | 0.15119 | 0.10516 |
| Kubusi | 0.045 | 0.04062 | 0.02495 | 0.03 | 0.01222 | 0.041 | - | 0.2119 |
| Fort Fordyce | 0.04469 | 0.05743 | 0.02274 | 0.03719 | 0.0342 | 0.055 | 0.03753 | - |

**Table S1.4** Pairwise *F*_ST_ and pairwise *D_PS_* (below and above diagonal, respectively) values for *P. stellata* (global *F_ST_* = 0.016, *p* < 0.001) based on eight microsatellite loci. * denotes significant *F_ST_* values (*p* <0.05)

|  | Ngele | Oribi Gorge | Gomo | Baziya | Manubi | Kubusi | Fort Fordyce | Alexandria | The Island |
| --- | --- | --- | --- | --- | --- | --- | --- | --- | --- |
| Ngele | - | 0.58242 | 0.6369 | 0.62946 | 0.62857 | 0.61905 | 0.62363 | 0.61765 | 0.60204 |
| Oribi Gorge | 0.0417 | - | 0.26923 | 0.23935 | 0.26374 | 0.26475 | 0.24176 | 0.31157 | 0.32575 |
| Gomo | **0.0207*** | 0.0214 | - | 0.16815 | 0.21786 | 0.2209 | 0.24908 | 0.28501 | 0.31122 |
| Baziya | **0.0278*** | **0.025** | **0.0274*** | - | 0.17083 | 0.19444 | 0.25172 | 0.26602 | 0.32079 |
| Manubi | **0.01869*** | 0.0307 | **0.0304*** | 0.029 | - | 0.17566 | 0.18608 | 0.22381 | 0.28299 |
| Kubusi | 0.01139 | 0.02014 | 0.02399 | 0.0251 | 0.0085 | - | 0.20014 | 0.19997 | 0.29403 |
| Fort Fordyce | **0.01847*** | 0.02557 | 0.02304 | 0.0226 | 0.00568 | 0.00832 | - | 0.22463 | 0.27355 |
| Alexandria | 0.0208 | 0.05298 | **0.03478*** | **0.01128*** | **0.03904*** | 0.01235 | **0.03156*** | - | 0.36375 |
| The Island | **0.03727*** | **0.0394*** | **0.04876*** | **0.01517*** | **0.04058*** | **0.01878*** | **0.0452*** | **0.0241*** | - |


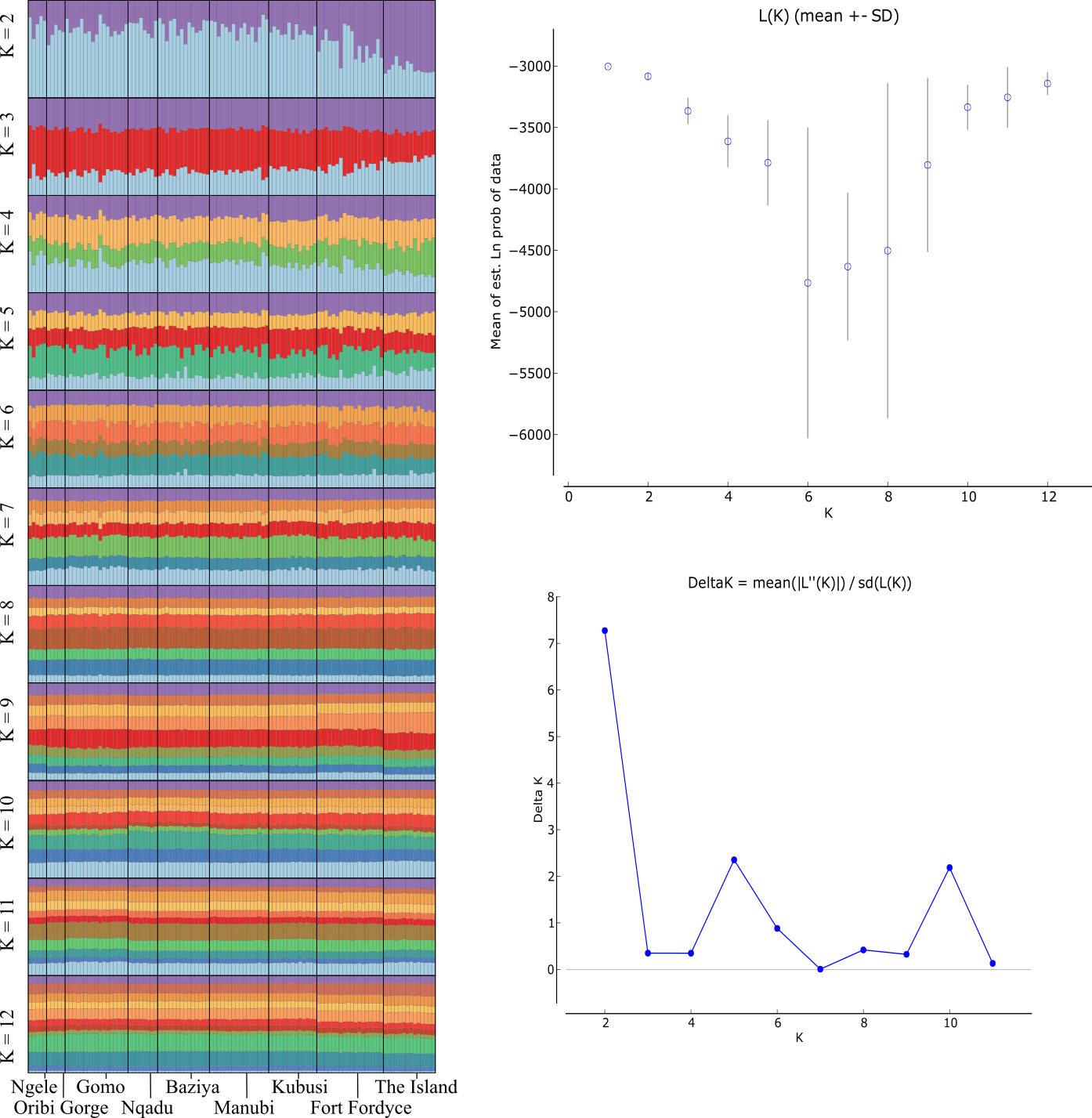


**Figure S1.1** (left) STRUCTURE Bayesian clustering exploration across 2-12 potential genetic clusters in *Batis capensis* individuals inhabiting nine forests across the Eastern Cape. (Top right) mean lnP(K) for each K value 2-12, with 95 % confidence intervals. (Bottom right) Evanno ΔK for each K value 2-12.


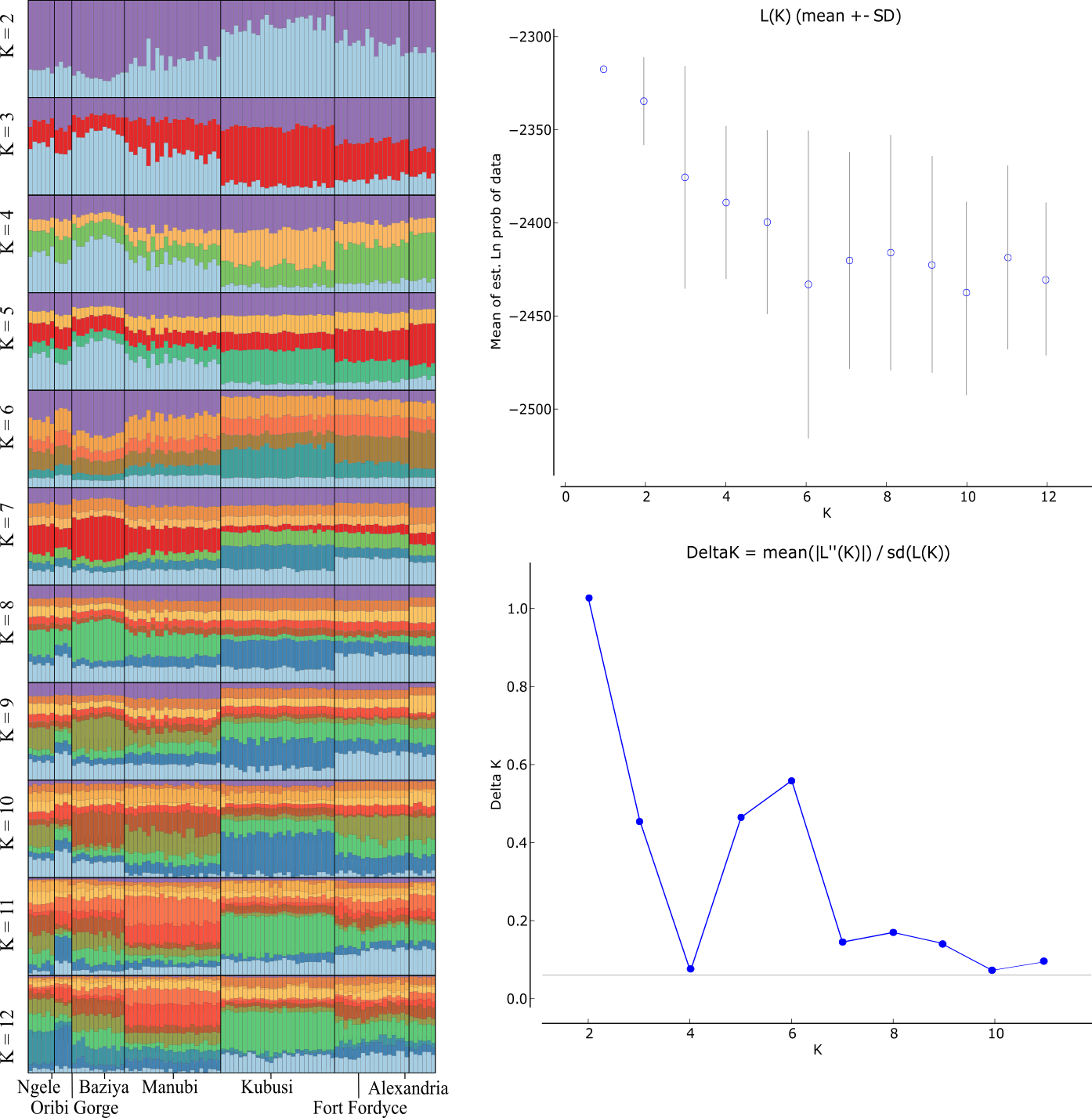


**Figure S1.2** (left) Bayesian clustering exploration across 2-12 potential genetic clusters in *Cossypha dichroa* individuals inhabiting seven forests across the Eastern Cape. (Top right) mean lnP(K) for each K value 2-12, with 95 % confidence intervals. (Bottom right) Evanno ΔK for each K value 2-12.


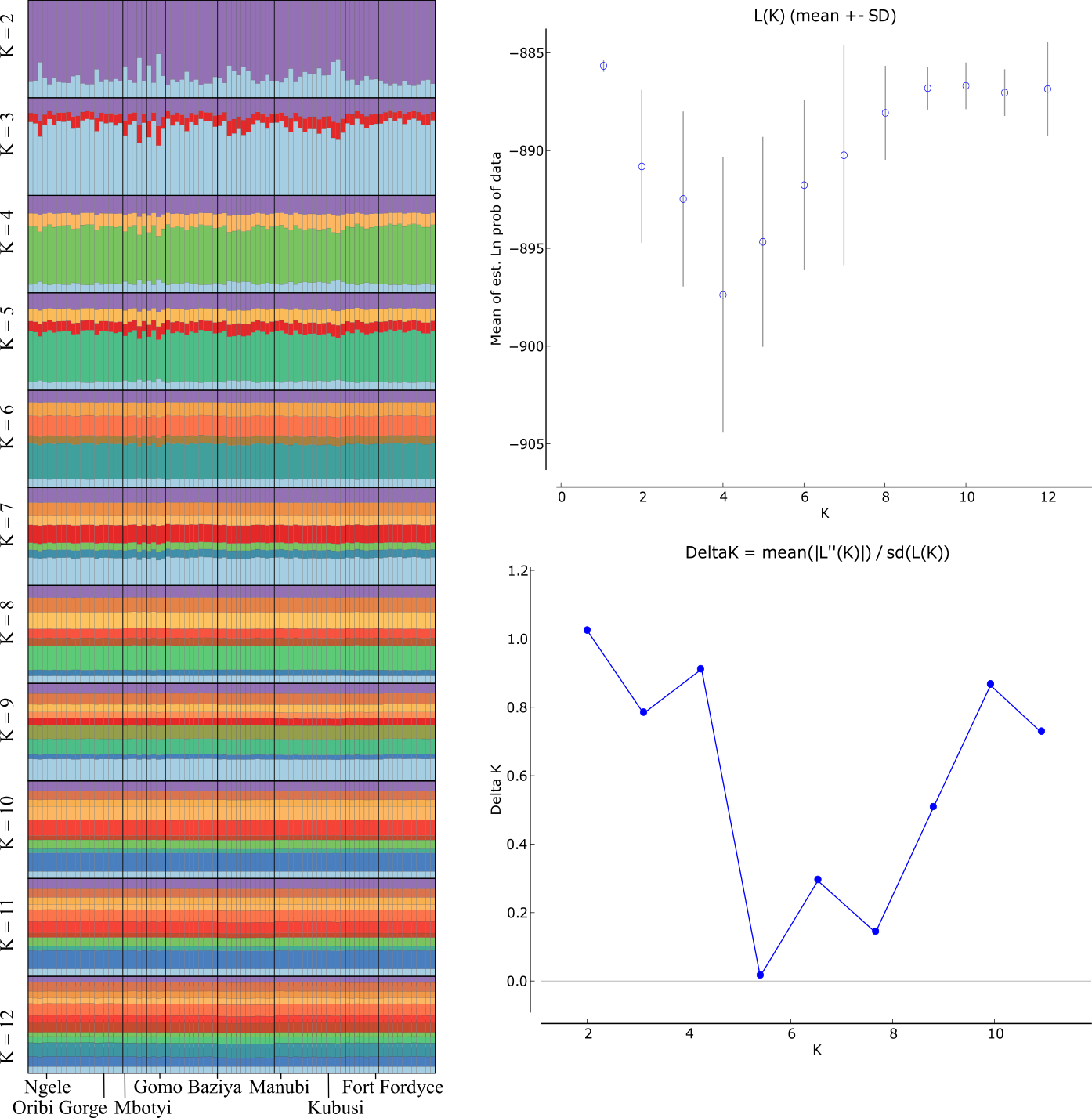


**Figure S1.3** (left) Bayesian clustering exploration across 2-12 potential genetic clusters in *Phylloscopus ruficapilla* individuals inhabiting seven forests across the Eastern Cape. (Top right) mean lnP(K) for each K value 2-12, with 95 % confidence intervals. (Bottom right) Evanno ΔK for each K value 2-12.


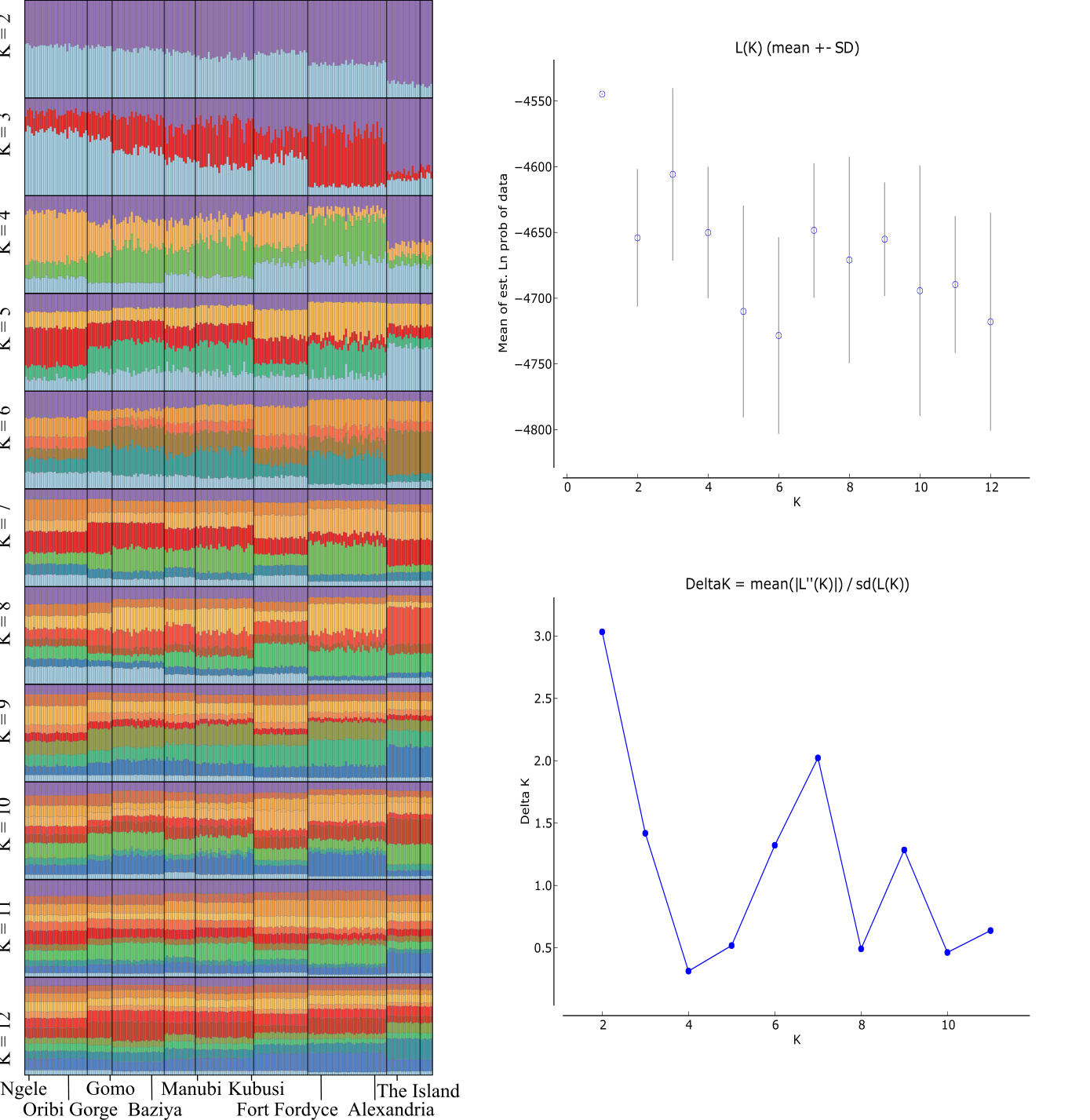


**Figure S1.4** (Left) Bayesian clustering exploration across 2-12 potential genetic clusters in *Pogonocichla stellata* individuals inhabiting seven forests across the Eastern Cape. (Top right) mean lnP(K) for each K value 2-12, with 95 % confidence intervals. (Bottom right) Evanno ΔK for each K value 2-12.

**Table S2** Estimated current effective populative sizes (*CN_e_*) for *B. capensis*, *C. dichroa*, *P. ruficapilla*, and *P. stellata* across a region of the Eastern Cape and southern KwaZulu Natal, inferred from eight species-specific microsatellite loci. Estimated *CN_e_* are for 1% and 2% critical allele frequencies, assuming both random mating and monogamy, with 95% confidence intervals (95% *CI*)

|  |  | *B. capensis* | |  | *C. dichroa* | |  | *P. ruficapilla* | |  | *P. stellata* | |
| --- | --- | --- | --- | --- | --- | --- | --- | --- | --- | --- | --- | --- |
|  |  | 0.02 | 0.01 |  | 0.02 | 0.01 |  | 0.02 | 0.01 |  | 0.02 | 0.01 |
| *CN_e_* Random |  | 340.6 | 342.3 |  | 238.8 | 358.8 |  | 831.7 | 332.1 |  | 424.3 | 515.3 |
| *95% CI* |  | 162.5-5497.9 | 169.1-2982.8 |  | 98.9-∞ | 137.8-∞ |  | 76.7-∞ | 71.5-∞ |  | 202.8-3902.9 | 240.1-11734.7 |
| *CN_e_* Monogamy |  | 682.7 | 686.0 |  | 479.1 | 719.0 |  | 1664.9 | 665.7 |  | 850.1 | 1032.1 |
| *95% CI* |  | 326.5-10997.5 | 339.7-5967.0 |  | 199.3-∞ | 277.0-∞ |  | 154.9-∞ | 144.5-∞ |  | 407.1-7807.2 | 481.6-23470.9 |


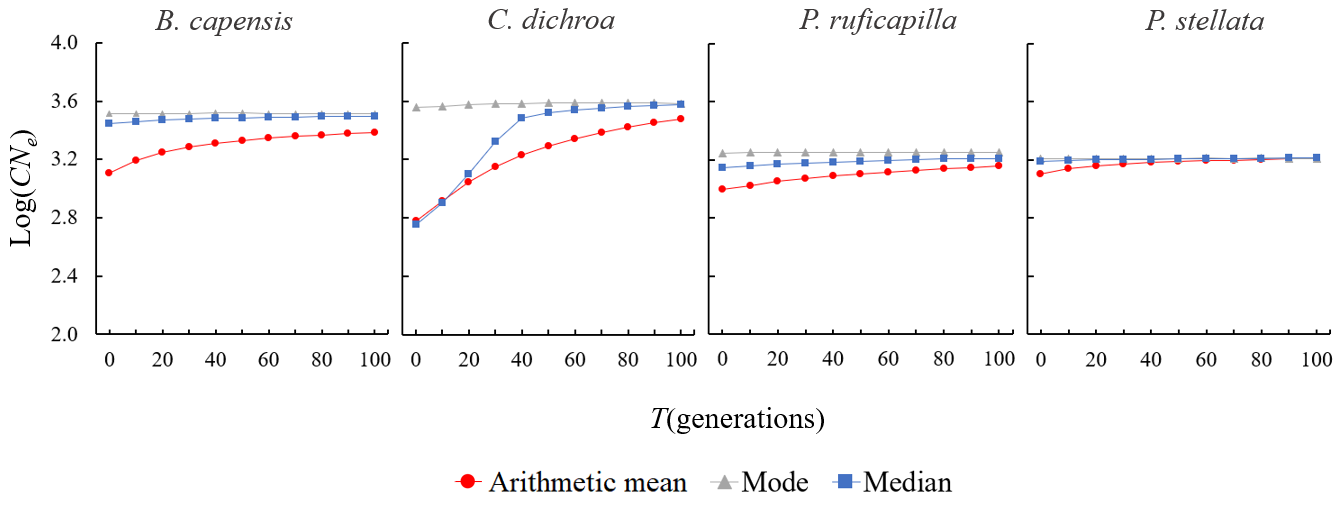


**Figure S2** VarEff plots showing variaion in *CN_e_* over the past 100 generations for *B. capensis*, *C. dichroa*, *P. ruficapilla*, and *P. stellata* among eleven forests across a region for the Eastern Cape and KwaZulu-Natal Provinces of South Africa, based respectively on eight microsatellites loci for each species, and assuming a two-phase (10% single-step) mutation model at a constant mutation rate of μ = 5x10^-4^ per generation.

**Figure S3** Relative performance of least cost pathway models based on landcover thematic surfaces for the four focal bird species, inferred from *D_PS_*. Positive ΔAICc values denote improved model performance over Euclidean distances alone.


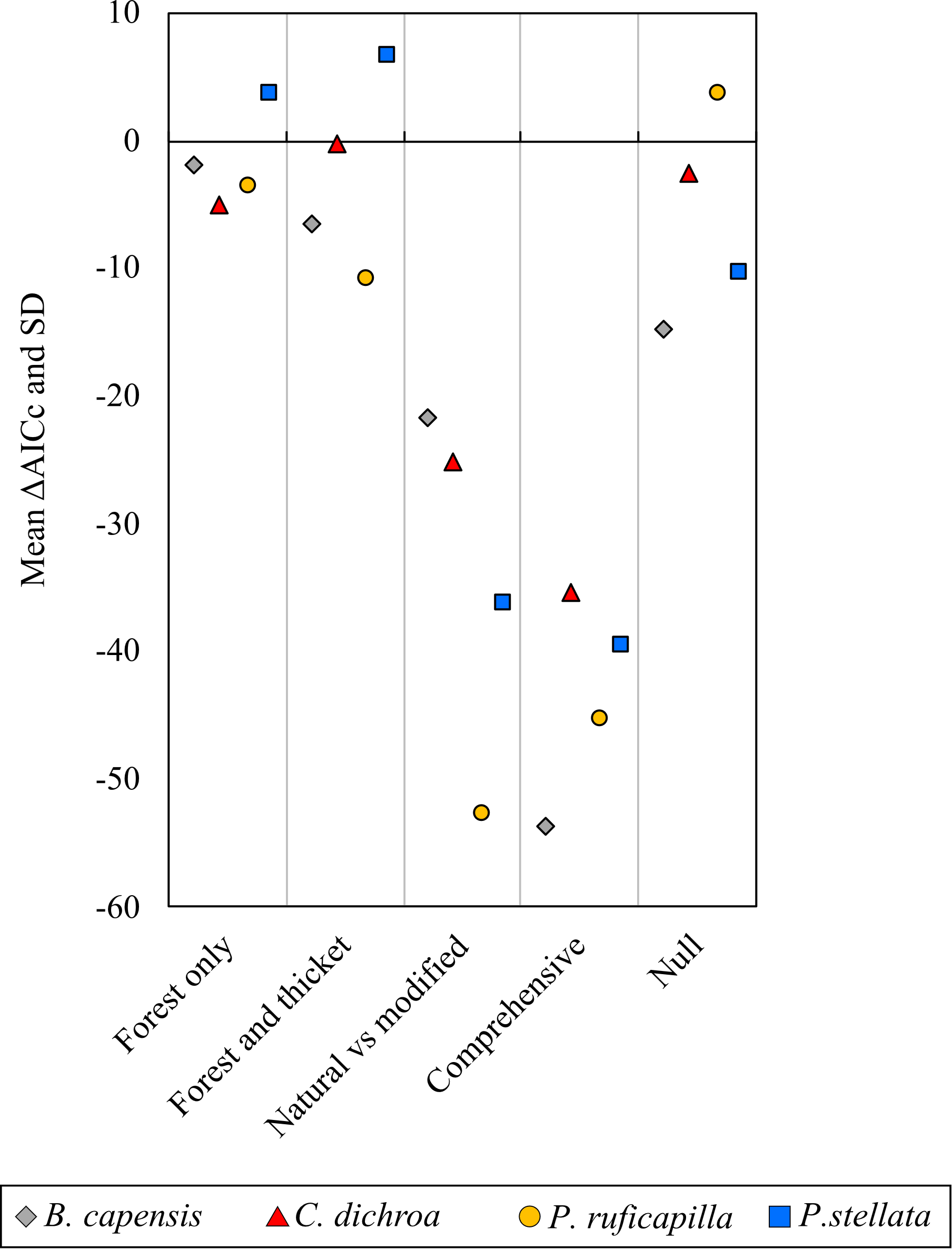

Supplement: Supplementary file 1 — Supplementary Material [file EVA-14-2680-s002.docx]
