# Supplementary material for: Species–landscape interactions drive divergent population trajectories in four forest‐dependent Afromontane forest songbird species within a biodiversity hotspot in South Africa
Source: Evol Appl. 2021 Oct 28;14(11):2680–97. doi: 10.1111/eva.13306 (PMC8591328; doi:10.1111/eva.13306)
Supplement: Supplementary file 2 — Appendix S1‐S3 [file EVA-14-2680-s001.docx]

**Title:** Species-landscape interactions drive divergent population trajectories in four forest-dependent Afromontane forest songbird species within a biodiversity hotspot in South Africa

**Appendix S1** procedures for polymerase chain reaction for amplifying the microsatellites used to genotype *Batis capensis*, *Cossypha dichroa*, *Phylloscopus ruficapilla*, and *Pogonocichla stellata*

Microsatellite sequences were amplified using polymerase chain reaction (PCR) in 10 μL reaction volumes containing approximately 50% (5 μL) KAPA2G Fast Multiplex Mix (© 2016 Kapa Biosystems) (1U *HotStart* DNA Polymerase; 1.5X KAPA2G Buffer A; 0.2 mM of each dNTP; 3.0 mM MgCl_2_); 0.1-0.6 μM of each forward and reverse primer (adjusted from 0.2 μM); and 20-50ng/μL template DNA. For each species, combinations of 3-5 microsatellite primer pairs were simultaneously amplified in multiplex PCRs in an GeneAmp PCR System 2700 thermocycler (® Applied Biosystems, USA), under the following PCR conditions: initial denaturation at 95°C for 3 min, followed by 25-35 cycles of 95°C for 15 s, T_anneal_ for 30 s, and 72°C for 25 s, with a final extension of 72°C of 2 min.

**Appendix S1.1** Microsatellite loci amplified in *B. capensis*

| Microsatellite | Reference | No. cycles | | T_anneal_ (°C) | Size (bp) |
| --- | --- | --- | --- | --- | --- |
| BMI-16 | (Wogan et al., 2016) | 25 | | 60 | 334-358 |
| BMI-17 | (Wogan et al., 2016) | 25 | | 60 | 148-204 |
| BMI-22 | (Wogan et al., 2016) | 25 | | 60 | 247-287 |
| BMI-44 | (Wogan et al., 2016) | 25 | | 60 | 144-164 |
| BMI-96 | (Wogan et al., 2016) | 25 | | 60 | 195-300 |
| BMI-41 | (Wogan et al., 2016) | 25 | | 60 | Monomorphic; 204 |
| BMI-71 | (Wogan et al., 2016) | 25 | | 60 | 136-192 |
| BMI-98 | (Wogan et al., 2016) | 25 | | 60 | 341-373 |
| BMI-32 | (Wogan et al., 2016) | 35 | | 60 | 304-332 |
|  |  |  | |  |  |
| **Appendix S1.2** Microsatellite loci amplified in *C. dichroa* (an additional 15 loci from Wogan et al. (2014), and 4 loci applied to *P. stellata* were screened but did not amplify) | | | | | |
| Microsatellite | Reference | | No. cycles | T_anneal_ (°C) | Size (bp) |
| CNA99 | (Wogan et al., 2014) | | 25 | 54 | 92-156 |
| CNA130 | (Wogan et al., 2014) | | 25 | 54 | 164-192 |
| CNA109 | (Wogan et al., 2014) | | 25 | 54 | 182-210 |
| CNA142 | (Wogan et al., 2014) | | 25 | 59 | 196-224 |
| WBSW9 | (McRae & Amos, 1999) | | 25 | 54 | 102-112 |
| Gf06 | (Petren, 1998) | | 25 | 59 | Stuttering; null alleles |
| CNA180 | (Wogan et al., 2014) | | 25 | 59 | 107-139 |
| CNA113 | (Wogan et al., 2014) | | 25 | 59 | 118-154 |
| CACA3 | (Wogan et al., 2014) | | 25 | 59 | 338-358 |
| CACA27 | (Wogan et al., 2014) | | 25 | 59 | Stuttering; null alleles |
|  |  | |  |  |  |
| **Appendix S1.3** Microsatellite loci amplified in *P. ruficapilla*. an additional 3 loci from Bensch et al. (1997) were screened but did not amplify) | | | | | |
| Microsatellite | Reference | | No. cycles | T_anneal_ (°C) | Size (bp) |
| Patmp2-43 | (Otter et al., 1998) | | 30 | 55 | 119-121 |
| Dpu-16 | (Dawson et al., 1997) | | 30 | 55 | 116-128 |
| MLSP4 | (Ishibashi et al., 2000) | | 30 | 55 | 147-155 |
| POCC8 | (Bensch et al., 1997) | | 30 | 55 | Null alleles; linked to POCC9 |
| POCC1 | (Bensch et al., 1997) | | 25 | 55 | 153-1533 |
| POCC5 | (Bensch et al., 1997) | | 25 | 55 | Monomorphic; 100 |
| POCC6 | (Bensch et al., 1997) | | 25 | 55 | 94-98 |
| POCC7 | (Bensch et al., 1997) | | 25 | 55 | 209-215 |
| POCC9 | (Bensch et al., 1997) | | 25 | 55 | 251-257 |
| Cu02* | (Gibbs et al., 1999) | | 35 | 57 | 151-169 |
| *amplified with cyclic extension period of 45 s | | | | | |
|  |  | |  |  |  |
| **Appendix S1.4** Microsatellite loci amplified in *P. stellata* (an additional 19 loci from (Wogan et al., 2014) were screened but did not amplify) | | | | | |
| Microsatellite | Reference | | No. cycles | T_anneal_ (°C) | Size (bp) |
| WBSW2* | (McRae & Amos, 1999) | | 25 | 55 | 121-123 |
| Ltmr6* | (McDonald & Potts, 1994) | | 25 | 55 | Monomorphic; 194 |
| Mcyu4* | (Double et al., 1997) | | 25 | 55 | 129-137 |
| Gf05* | (Petren, 1998) | | 25 | 55 | Stuttering; null alleles |
| CNA142* | (Wogan et al., 2014) | | 25 | 55 | 199-243 |
| CNA162 | (Wogan et al., 2014) | | 35 | 59 | 228-276 |
| CACA3 | (Wogan et al., 2014) | | 35 | 59 | 312-352 |
| CACA27 | (Wogan et al., 2014) | | 35 | 59 | 330-368 |
| Gf06 | (Petren, 1998) | | 35 | 59 | 106-116 |
| WBSW9 | (McRae & Amos, 1999) | | 30 | 50 | 141-159 |
| * reactions performed with 5% Dimethyl sulfoxide (DMSO) | | | | | |

# Appendix S1 references

Bensch, S., Price, T., & Kohn, J. (1997). Isolation and characterization of microsatellite loci in a Phylloscopus warbler. *Molecular Ecology*. doi: 10.1046/j.1365-294X.1997.00150.x

Dawson, R. J. G., Gibbs, H. L., Hobson, K. A., & Yezerinac, S. M. (1997). Isolation of microsatellite DNA markers from a passerine bird, Dendroica petechia (the yellow warbler), and their use in population studies. *Heredity*. doi: 10.1038/hdy.1997.190

Double, M. C., Dawson, D., Burke, T., & Cockburn, A. (1997). Finding the fathers in the least faithful bird: A microsatellite-based genotyping system for the superb fairy-wren Malurus cyaneus. *Molecular Ecology*. doi: 10.1046/j.1365-294X.1997.00228.x

Gibbs, L. H., Tabak, L. M., & Hobson, K. (1999). Characterization of microsatellite DNA loci for a neotropical migrant songbird, the Swainson’s thrush (Catharus ustulatus). *Molecular Ecology*. doi: 10.1046/j.1365-294X.1999.00673.x

Ishibashi, Y., Mikami, O., & Abe, S. (2000). Isolation and characterization of microsatellite loci in the Japanese marsh warbler Locustella pryeri. *Molecular Ecology*. doi: 10.1046/j.1365-294X.2000.00874-5.x

McDonald, D. B., & Potts, W. K. (1994). Cooperative display and relatedness among males in a lek-mating bird. *Science*, *266*(5187), 1030–1032. doi: 10.1126/science.7973654

McRae, B., & Amos, W. (1999). Characterization of hypervariable microsatellites in the cooperatively breeding white-browed sparrow weaver Plocepasser mahali. *Molecular Ecology*, *8*(5), 803.

Otter, K., Ratcliffe, L., Michaud, D., & Boag, P. T. (1998). Do female black-capped chickadees prefer high-ranking males as extra-pair partners? *Behavioral Ecology and Sociobiology*. doi: 10.1007/s002650050463

Petren, K. (1998). Microsatellite primers from Geospiza fortis and cross-species amplification in Darwin’s finches. *Molecular Ecology*. doi: 10.1046/j.1365-294x.1998.00518.x

Wogan, G. O. U., Feldheim, K. A., Tsai, A. S., Brown, A. A., Kapelke, J., Galinato, M., … Bowie, R. C. K. (2016). New genetic resources and a preliminary multi-locus assessment of species boundaries in the Batis capensis species complex (Passeriformes: Platysteridae). *Biochemical Systematics and Ecology*, *65*, 83–88. doi: 10.1016/j.bse.2016.01.007

Wogan, G. O. U., Feldheim, K. A., Voelker, G., & Bowie, R. C. K. (2014). Development and characterization of thirteen microsatellite markers for the Fiscal Flycatcher (Sigelus silens) for use in phylogeographic and landscape genetics research. *Conservation Genetics Resources*, *7*(1), 125–127. doi: 10.1007/s12686-014-0309-2

# Appendix S2: Microsatellite locus diversity

**Appendix S2.1** Microsatellite locus genetic diversity measures for *B. capensis*: *N* – number of individuals; *NA* – number of alleles; *AR* – allelic richness; *HO* – observed heterozygosity; *HE* – expected heterozygosity; *NULL* – null allele frequency; *F_IS_* – inbreeding coefficient. *Significant deviations to Hardy–Weinberg equilibrium after Benjamini-Hochberg(*p* < 0.05)

|  | Ngele | Oribi Gorge | Gomo | Nqadu | Baziya | Manubi | Kubusi | Fort Fordyce | The Island | Overall |
| --- | --- | --- | --- | --- | --- | --- | --- | --- | --- | --- |
|  | *BMI-16* | | | | | | | | | |
| *N* | 6 | 8 | 17 | 8 | 14 | 16 | 13 | 18 | 14 | 114 |
| *NA* | 2 | 3 | 3 | 1 | 3 | 5 | 2 | 4 | 4 | 7 |
| *AR* | 1.94 | 2.58 | 1.55 | 1 | 1.85 | 2.33 | 1.83 | 1.86 | 2.16 | 1.90 |
| *Ho* | 0.40 | 0.40 | 0.12 | 0 | 0.07 | 0.25 | 0.23 | 0.18 | 0.27 | 0.21 |
| *He* | 0.32 | 0.54 | 0.16 | 0 | 0.31 | 0.33 | 0.31 | 0.29 | 0.42 | 0.30 |
| *NULL* | 0.00 | 0.09 | 0.00 | 0.05 | 0.00 | 0.03 | 0.04 | 0.02 | 0.11 | 0.07 |
| *F_IS_* | -0.14 | 0.36 | 0.00 | 0.78 | 0.00 | 0.27 | 0.29 | 0.63 | 0.84 | 0.50 |
|  | *BMI-17* | | | | | | | | | |
| *N* | 6 | 8 | 17 | 8 | 14 | 16 | 13 | 18 | 14 | 114 |
| *NA* | 5 | 4 | 7 | 6 | 6 | 7 | 8 | 8 | 7 | 15 |
| *AR* | 3.97 | 3.33 | 4.63 | 4.57 | 4.43 | 4.23 | 4.99 | 4.58 | 4.76 | 4.39 |
| *Ho* | 0.80 | 0.60 | 0.82 | 0.88 | 0.71 | 0.54 | 0.85 | 0.78 | 0.79 | 0.75 |
| *He* | 0.68 | 0.66 | 0.79 | 0.8 | 0.78 | 0.68 | 0.81 | 0.78 | 0.80 | 0.75 |
| *NULL* | 0.00 | 0.00 | 0.00 | 0.02 | 0.00 | 0.00 | 0.00 | 0.00 | 0.00 | 0.04 |
| *F_IS_* | -0.07 | 0.20 | -0.02 | 0.12 | -0.03 | 0.46 | 0.00 | 0.04 | 0.06 | 0.10 |
|  | *BMI-22* | | | | | | | | | |
| *N* | 6 | 8 | 17 | 8 | 14 | 16 | 13 | 18 | 14 | 114 |
| *NA* | 6 | 5 | 9 | 7 | 8 | 9 | 9 | 7 | 8 | 11 |
| *AR* | 4.64 | 4.00 | 4.79 | 5.04 | 5.07 | 5.25 | 5.10 | 4.87 | 5.24 | 4.89 |
| *Ho* | 1.00 | 0.80 | 0.88 | 0.88 | 0.86 | 0.81 | 0.92 | 0.83 | 0.79 | 0.86 |
| *He* | 0.78 | 0.72 | 0.78 | 0.82 | 0.81 | 0.83 | 0.80 | 0.81 | 0.84 | 0.80 |
| *NULL* | 0.00 | 0.00 | 0.00 | 0 | 0.00 | 0.00 | 0.00 | 0.00 | 0.02 | 0.00 |
| *F_IS_* | -0.18 | 0.00 | -0.10 | -0.02 | 0.00 | 0.06 | -0.12 | 0.00 | 0.10 | -0.02 |
|  | *BMI-44* | | | | | | | | | |
| *N* | 6 | 8 | 17 | 8 | 14 | 16 | 13 | 18 | 14 | 114 |
| *NA* | 1 | 2 | 3 | 3 | 2 | 2 | 2 | 2 | 2 | 6 |
| *AR* | 1.00 | 1.99 | 2.21 | 2.54 | 1.72 | 1.76 | 1.91 | 1.92 | 1.97 | 1.89 |
| *Ho* | 0.00 | 0.60 | 0.41 | 0.38 | 0.14 | 0.25 | 0.31 | 0.33 | 0.43 | 0.32 |
| *He* | 0.00 | 0.42 | 0.41 | 0.48 | 0.24 | 0.22 | 0.36 | 0.35 | 0.41 | 0.32 |
| *NULL* | 0.00 | 0.00 | 0.00 | 0.11 | 0.09 | 0.00 | 0.04 | 0.01 | 0.00 | 0.05 |
| *F_IS_* | 0.00 | -0.33 | -0.28 | 0.45 | 0.28 | -0.11 | 0.17 | 0.06 | 0.01 | 0.03 |

**Appendix S2.1** (continued)

|  | Ngele | Oribi Gorge | Gomo | Nqadu | Baziya | Manubi | Kubusi | Fort Fordyce | The Island | Overall |
| --- | --- | --- | --- | --- | --- | --- | --- | --- | --- | --- |
|  | *BMI-96* | | | | | | | | | |
| *N* | 6 | 8 | 17 | 8 | 14 | 16 | 13 | 18 | 14 | 114 |
| *NA* | 7 | 6 | 11 | 11 | 12 | 17 | 12 | 14 | 9 | 22 |
| *AR* | 5.28 | 4.83 | 6.00 | 6.52 | 6.43 | 7.31 | 6.46 | 6.70 | 5.36 | 6.10 |
| *Ho* | 1.00 | 1.00 | 0.94 | 1 | 1.00 | 1.00 | 0.92 | 0.94 | 0.86 | 0.96 |
| *He* | 0.82 | 0.80 | 0.86 | 0.89 | 0.88 | 0.92 | 0.89 | 0.90 | 0.83 | 0.87 |
| *NULL* | 0.00 | 0.00 | 0.00 | 0 | 0.00 | 0.00 | 0.00 | 0.00 | 0.00 | 0.00 |
| *F_IS_* | -0.11 | -0.14 | -0.07 | -0.1 | -0.06 | -0.06 | 0.00 | -0.03 | 0.01 | -0.05 |
|  | *BMI-32* | | | | | | | | | |
| *N* | 6 | 8 | 17 | 8 | 14 | 16 | 13 | 18 | 14 | 114 |
| *NA* | 8 | 6 | 9 | 6 | 7 | 9 | 9 | 8 | 7 | 15 |
| *AR* | 5.61 | 4.62 | 5.15 | 4.56 | 4.70 | 5.64 | 5.39 | 5.53 | 5.06 | 5.14 |
| *Ho* | 0.80 | 0.80 | 0.71 | 0.62 | 0.79 | 0.88 | 0.77 | 0.83 | 0.86 | 0.78 |
| *He* | 0.86 | 0.80 | 0.81 | 0.82 | 0.79 | 0.86 | 0.85 | 0.85 | 0.81 | 0.83 |
| *NULL* | 0.00 | 0.00 | 0.03 | 0 | 0.12 | 0.00 | 0.05 | 0.02 | 0.00 | 0.04 |
| *F_IS_* | 0.18 | 0.11 | 0.15 | 0.04 | 0.30 | 0.01 | 0.14 | 0.05 | -0.02 | 0.09 |
|  | *BMI-98* | | | | | | | | | |
| *N* | 6 | 8 | 17 | 8 | 14 | 16 | 13 | 18 | 14 | 114 |
| *NA* | 5 | 5 | 7 | 6 | 8 | 6 | 5 | 7 | 4 | 9 |
| *AR* | 4.18 | 4.16 | 4.29 | 3.87 | 4.73 | 4.45 | 4.12 | 4.66 | 3.78 | 4.25 |
| *Ho* | 0.80 | 0.80 | 0.59 | 0.5 | 0.79 | 0.81 | 0.92 | 0.72 | 0.79 | 0.75 |
| *He* | 0.78 | 0.76 | 0.76 | 0.73 | 0.73 | 0.74 | 0.76 | 0.80 | 0.74 | 0.76 |
| *NULL* | 0.00 | 0.00 | 0.08 | 0 | 0.15* | 0.00 | 0.00 | 0.02 | 0.00 | 0.01 |
| *F_IS_* | 0.09 | 0.06 | 0.25 | -0.04 | 0.38 | -0.07 | -0.17 | 0.12 | -0.02 | 0.06 |
|  | *BMI-71* | | | | | | | | | |
| *N* | 6 | 8 | 17 | 8 | 14 | 16 | 13 | 18 | 14 | 114 |
| *NA* | 4 | 3 | 8 | 5 | 6 | 6 | 4 | 5 | 4 | 8 |
| *AR* | 3.25 | 2.35 | 3.98 | 3.09 | 3.62 | 3.65 | 2.77 | 3.60 | 3.32 | 3.29 |
| *Ho* | 0.60 | 0.33 | 0.41 | 0.57 | 0.59 | 0.59 | 0.45 | 0.52 | 0.59 | 0.52 |
| *He* | 0.64 | 0.46 | 0.73 | 0.75 | 0.76 | 0.79 | 0.66 | 0.76 | 0.70 | 0.69 |
| *NULL* | 0.02 | 0.02 | 0.09 | 0.03 | 0.03 | 0.34 | 0.03 | 0.03 | 0.04 | 0.05 |
| *F_IS_* | 0.17 | 0.14 | 0.36 | 0.15 | 1.00* | 0.78 | 0.18 | 0.12 | 0.22 | 0.47 |

**Appendix S2.2** Microsatellite locus genetic diversity measures for *C. dichroa*: *N* – number of individuals; *NA* – number of alleles; *AR* – allelic richness; *HO* – observed heterozygosity; *HE* – expected heterozygosity; *NULL* – null allele frequency; *F_IS_* – inbreeding coefficient. *Significant deviations to Hardy–Weinberg equilibrium after Benjamini-Hochberg(*p* < 0.05)

|  | Ngele | Oribi Gorge | Baziya | Manubi | Kubusi | Fort Fordyce | Alexandria | Overall |
| --- | --- | --- | --- | --- | --- | --- | --- | --- |
|  | *WBSW9* | | | | | | | |
| *N* | 6 | 5 | 12 | 22 | 26 | 17 | 6 | 94 |
| *NA* | 5 | 2 | 2 | 6 | 6 | 3 | 5 | 6 |
| *AR* | 3.36 | 1.70 | 1.30 | 2.71 | 3.47 | 1.89 | 3.77 | 2.60 |
| *Ho* | 0.83 | 0.25 | 0.08 | 0.41 | 0.58 | 0.24 | 0.83 | 0.46 |
| *He* | 0.61 | 0.22 | 0.08 | 0.48 | 0.67 | 0.29 | 0.75 | 0.44 |
| *NULL* | 0.00 | 0.00 | 0.00 | 0.02 | 0.05 | 0.05 | 0.00 | 0.05 |
| *F_IS_* | -0.28 | 0.00 | 0.00 | 0.17 | 0.22 | 0.24 | -0.02 | 0.13 |
|  | *CNA130* | | | | | | | |
| *N* | 6 | 5 | 12 | 22 | 26 | 17 | 6 | 94 |
| *NA* | 4 | 4 | 4 | 8 | 6 | 5 | 4 | 8 |
| *AR* | 3.36 | 3.38 | 3.38 | 4.41 | 3.72 | 3.60 | 3.04 | 3.56 |
| *Ho* | 1.00 | 1.00 | 0.59 | 0.86 | 0.73 | 0.88 | 0.83 | 0.84 |
| *He* | 0.68 | 0.69 | 0.73 | 0.79 | 0.73 | 0.71 | 0.62 | 0.71 |
| *NULL* | 0.00 | 0.00 | 0.06 | 0.00 | 0.00 | 0.00 | 0.00 | 0.00 |
| *F_IS_* | -0.40 | -0.33 | 0.24 | -0.08 | 0.02 | -0.21 | -0.25 | -0.07 |
|  | *CNA109* | | | | | | | |
| *N* | 6 | 5 | 12 | 22 | 26 | 17 | 6 | 94 |
| *NA* | 3 | 4 | 5 | 6 | 6 | 4 | 4 | 8 |
| *AR* | 2.71 | 3.14 | 3.64 | 3.56 | 3.50 | 2.88 | 3.20 | 3.23 |
| *Ho* | 0.50 | 0.50 | 0.67 | 0.82 | 0.81 | 0.59 | 0.50 | 0.63 |
| *He* | 0.62 | 0.56 | 0.70 | 0.72 | 0.70 | 0.62 | 0.68 | 0.66 |
| *NULL* | 0.06 | 0.00 | 0.00 | 0.00 | 0.00 | 0.02 | 0.08 | 0.02 |
| *F_IS_* | 0.29 | 0.05 | 0.10 | -0.12 | -0.13 | 0.08 | 0.35 | 0.01 |
|  | *CNA99* | | | | | | | |
| *N* | 6 | 5 | 12 | 22 | 26 | 17 | 6 | 94 |
| *NA* | 6 | 6 | 9 | 12 | 13 | 7 | 4 | 17 |
| *AR* | 3.92 | 4.46 | 4.59 | 4.82 | 4.85 | 4.04 | 3.25 | 4.28 |
| *Ho* | 0.83 | 0.75 | 0.83 | 0.82 | 0.73 | 0.82 | 0.67 | 0.78 |
| *He* | 0.69 | 0.78 | 0.80 | 0.82 | 0.83 | 0.75 | 0.69 | 0.77 |
| *NULL* | 0.00 | 0.00 | 0.00 | 0.00 | 0.04 | 0.00 | 0.01 | 0.01 |
| *F_IS_* | -0.11 | 0.18 | 0.00 | 0.03 | 0.14 | -0.06 | 0.1304 | 0.05 |

**Appendix S2.2** (continued)

|  | Ngele | Oribi Gorge | Baziya | Manubi | Kubusi | Fort Fordyce | Alexandria | Overall |
| --- | --- | --- | --- | --- | --- | --- | --- | --- |
|  | *CNA142* | | | | | | | |
| *N* | 6 | 5 | 12 | 22 | 26 | 17 | 6 | 94 |
| *NA* | 4 | 3 | 6 | 8 | 6 | 7 | 3 | 8 |
| *AR* | 3.23 | 2.36 | 4.14 | 4.22 | 3.95 | 4.45 | 2.77 | 3.59 |
| *Ho* | 0.67 | 0.50 | 0.75 | 0.91 | 0.77 | 0.76 | 0.67 | 0.72 |
| *He* | 0.65 | 0.41 | 0.79 | 0.75 | 0.76 | 0.81 | 0.61 | 0.68 |
| *NULL* | 0.00 | 0.00 | 0.03 | 0.00 | 0.00 | 0.01 | 0.00 | 0.03 |
| *F_IS_* | 0.07 | -0.09 | 0.10 | -0.19 | 0.01 | 0.09 | 0.00 | 0.01 |
|  | *CNA113* | | | | | | | |
| *N* | 6 | 5 | 12 | 22 | 26 | 17 | 6 | 94 |
| *NA* | 3 | 3 | 6 | 9 | 8 | 9 | 4 | 10 |
| *AR* | 2.74 | 2.32 | 3.41 | 4.34 | 4.50 | 4.72 | 2.95 | 3.57 |
| *Ho* | 0.67 | 0.25 | 0.83 | 0.82 | 0.81 | 0.88 | 0.83 | 0.73 |
| *He* | 0.61 | 0.41 | 0.61 | 0.79 | 0.81 | 0.82 | 0.58 | 0.66 |
| *NULL* | 0.00 | 0.00 | 0.00 | 0.00 | 0.00 | 0.00 | 0.00 | 0.00 |
| *F_IS_* | 0.00 | 0.50 | -0.33 | -0.01 | 0.02 | -0.05 | -0.35 | -0.04 |
|  | *CACA3* | | | | | | | |
| *N* | 6 | 5 | 12 | 22 | 26 | 17 | 6 | 94 |
| *NA* | 3 | 1 | 4 | 4 | 5 | 4 | 2 | 6 |
| *AR* | 2.33 | 1.00 | 2.79 | 2.37 | 2.69 | 2.10 | 1.50 | 2.11 |
| *Ho* | 0.50 | 0.00 | 0.58 | 0.36 | 0.42 | 0.35 | 0.17 | 0.34 |
| *He* | 0.40 | 0.00 | 0.55 | 0.41 | 0.44 | 0.31 | 0.15 | 0.32 |
| *NULL* | 0.00 | 0.00 | 0.00 | 0.04 | 0.00 | 0.00 | 0.00 | 0.00 |
| *F_IS_* | -0.15 | 0.00 | -0.02 | 0.14 | 0.07 | -0.12 | 0.00 | 0.0242 |
|  | *CNA180* | | | | | | | |
| *N* | 6 | 5 | 12 | 22 | 26 | 17 | 6 | 94 |
| *NA* | 4 | 2 | 4 | 7 | 6 | 7 | 3 | 9 |
| *AR* | 2.79 | 1.72 | 2.98 | 3.31 | 3.52 | 3.48 | 2.79 | 2.94 |
| *Ho* | 0.50 | 0.25 | 0.75 | 0.68 | 0.81 | 0.53 | 0.83 | 0.62 |
| *He* | 0.51 | 0.22 | 0.62 | 0.66 | 0.70 | 0.65 | 0.61 | 0.57 |
| *NULL* | 0.00 | 0.00 | 0.00 | 0.03 | 0.02 | 0.05 | 0.00 | 0.02 |
| *F_IS_* | 0.12 | 0.00 | -0.16 | -0.01 | -0.14 | 0.21 | -0.28 | -0.04 |

**Appendix S2.3** Microsatellite locus genetic diversity measures for *P. ruficapilla*: *N* – number of individuals; *NA* – number of alleles; *AR* – allelic richness; *HO* – observed heterozygosity; *HE* – expected heterozygosity; *NULL* – null allele frequency; *F_IS_* – inbreeding coefficient. *Significant deviations to Hardy–Weinberg equilibrium after Benjamini-Hochberg(*p* < 0.05)

|  | Ngele | Oribi Gorge | | Mbotyi | | Gomo | | Baziya | | Manubi | | Kubusi | | Fort Fordyce | | Overall | |
| --- | --- | --- | --- | --- | --- | --- | --- | --- | --- | --- | --- | --- | --- | --- | --- | --- | --- |
|  | *Pat-43* | | | | | | | | | | | | | | | | |
| N | 20 | | 6 | | 6 | | 11 | | 14 | | 15 | | 9 | | 11 | | 92 |
| *NA* | 2 | | 2 | | 2 | | 2 | | 2 | | 2 | | 2 | | 2 | | 2 |
| *AR* | 1.99 | | 2.00 | | 1.99 | | 2.00 | | 1.87 | | 1.99 | | 1.77 | | 1.98 | | 1.95 |
| *Ho* | 0.60 | | 0.60 | | 0.43 | | 0.45 | | 0.25 | | 0.47 | | 0.25 | | 0.45 | | 0.44 |
| *He* | 0.45 | | 0.42 | | 0.50 | | 0.50 | | 0.33 | | 0.46 | | 0.22 | | 0.43 | | 0.41 |
| *NULL* | 0.00 | | 0.00 | | 0.00 | | 0.03 | | 0.07 | | 0.00 | | 0.00 | | 0.00 | | 0.00 |
| *F_IS_* | -0.30 | | -0.33 | | 0.5714 | | 0.14 | | 0.28 | | 0.03 | | -0.07 | | 0.00 | | 0.00 |
|  | *MLSP4* | | | | | | | | | | | | | | | | |
| N | 20 | | 6 | | 6 | | 11 | | 14 | | 15 | | 9 | | 11 | | 92 |
| *NA* | 5 | | 2 | | 4 | | 2 | | 3 | | 4 | | 4 | | 3 | | 7 |
| *AR* | 1.94 | | 1.68 | | 3.45 | | 1.37 | | 2.22 | | 2.11 | | 2.88 | | 2.21 | | 2.23 |
| *Ho* | 0.20 | | 0.20 | | 0.83 | | 0.09 | | 0.25 | | 0.27 | | 0.50 | | 0.36 | | 0.34 |
| *He* | 0.19 | | 0.18 | | 0.65 | | 0.09 | | 0.39 | | 0.24 | | 0.54 | | 0.31 | | 0.32 |
| *NULL* | 0.00 | | 0.00 | | 0.00 | | 0.00 | | 0.09 | | 0.00 | | 0.01 | | 0.00 | | 0.02 |
| *F_IS_* | -0.04 | | 0.00 | | 0.00 | | 0.00 | | 0.40 | | -0.07 | | 0.07 | | -0.13 | | 0.06 |
|  | *Dpu-16* | | | | | | | | | | | | | | | | |
| N | 20 | | 6 | | 6 | | 11 | | 14 | | 15 | | 9 | | 11 | | 92 |
| *NA* | 3 | | 2 | | 3 | | 3 | | 3 | | 3 | | 3 | | 2 | | 5 |
| *AR* | 1.98 | | 1.68 | | 2.19 | | 1.78 | | 2.45 | | 1.82 | | 1.96 | | 1.63 | | 1.94 |
| *Ho* | 0.30 | | 0.20 | | 0.33 | | 0.18 | | 0.50 | | 0.20 | | 0.25 | | 0.18 | | 0.27 |
| *He* | 0.26 | | 0.18 | | 0.29 | | 0.17 | | 0.40 | | 0.18 | | 0.23 | | 0.17 | | 0.24 |
| *NULL* | 0.00 | | 0.00 | | 0.00 | | 0.00 | | 0.00 | | 0.00 | | 0.00 | | 0.00 | | 0.00 |
| *F_IS_* | -0.12 | | 0.00 | | 0.00 | | -0.03 | | -0.20 | | -0.05 | | -0.03 | | -0.05 | | -0.10 |
|  | *Cu02* | | | | | | | | | | | | | | | | |
| N | 20 | | 6 | | 6 | | 11 | | 14 | | 15 | | 9 | | 11 | | 92 |
| *NA* | 4 | | 2 | | 2 | | 2 | | 3 | | 3 | | 2 | | 2 | | 10 |
| *AR* | 2.43 | | 1.67 | | 1.97 | | 2.00 | | 2.35 | | 2.28 | | 2.00 | | 2.00 | | 2.09 |
| *Ho* | 0.55 | | 0.20 | | 0.52 | | 0.73 | | 0.58 | | 0.53 | | 0.62 | | 0.64 | | 0.55 |
| *He* | 0.54 | | 0.18 | | 0.49 | | 0.50 | | 0.53 | | 0.53 | | 0.49 | | 0.48 | | 0.47 |
| *NULL* | 0.00 | | 0.00 | | 0.00 | | 0.00 | | 0.00 | | 0.00 | | 0.00 | | 0.00 | | 0.00 |
| *F_IS_* | 0.00 | | 0.00 | | 0.57 | | -0.43 | | -0.05 | | 0.02 | | -0.05 | | -0.27 | | -0.07 |

**Appendix S2.3** (continued)

|  | Ngele | Oribi Gorge | Mbotyi | Gomo | Baziya | Manubi | Kubusi | Fort Fordyce | Overall |
| --- | --- | --- | --- | --- | --- | --- | --- | --- | --- |
|  | *POCC9* | | | | | | | | |
| N | 20 | 6 | 6 | 11 | 14 | 15 | 9 | 11 | 92 |
| *NA* | 4 | 4 | 4 | 3 | 3 | 4 | 2 | 1 | 4 |
| *AR* | 2.21 | 3.04 | 3.42 | 2.31 | 2.13 | 2.38 | 1.48 | 1.00 | 2.25 |
| *Ho* | 0.30 | 0.43 | 0.67 | 0.45 | 0.33 | 0.39 | 0.12 | 0.00 | 0.34 |
| *He* | 0.27 | 0.58 | 0.65 | 0.37 | 0.29 | 0.46 | 0.12 | 0.00 | 0.34 |
| *NULL* | 0.00 | 0.03 | 0.00 | 0.00 | 0.00 | 0.00 | 0.00 | 0.00 | 0.00 |
| *F_IS_* | -0.09 | 0.71 | 0.25 | -0.19 | -0.11 | 0.59 | 0.00 | 0.00 | 0.21 |
|  | *POCC6* | | | | | | | | |
| N | 20 | 6 | 6 | 11 | 14 | 15 | 9 | 11 | 92 |
| *NA* | 2 | 2 | 2 | 2 | 2 | 2 | 2 | 2 | 2 |
| *AR* | 1.76 | 1.99 | 1.96 | 1.89 | 1.87 | 1.95 | 1.78 | 1.90 | 1.89 |
| *Ho* | 0.20 | 0.60 | 0.17 | 0.36 | 0.25 | 0.33 | 0.25 | 0.27 | 0.30 |
| *He* | 0.26 | 0.42 | 0.49 | 0.30 | 0.33 | 0.42 | 0.22 | 0.35 | 0.35 |
| *NULL* | 0.06 | 0.00 | 0.22 | 0.00 | 0.07 | 0.07 | 0.00 | 0.07 | 0.04 |
| *F_IS_* | 0.24 | -0.33 | 0.57 | -0.18 | 0.28 | 0.24 | -0.07 | 0.27 | 0.17 |
|  | *POCC7* | | | | | | | | |
| N | 20 | 6 | 6 | 11 | 14 | 15 | 9 | 11 | 92 |
| *NA* | 2 | 2 | 2 | 2 | 2 | 3 | 2 | 2 | 3 |
| *AR* | 2 | 1.99 | 2 | 2 | 2 | 2.29 | 2 | 1.99 | 2.12 |
| *Ho* | 0.8 | 0.4 | 0.67 | 0.55 | 0.58 | 0.6 | 0.5 | 0.55 | 0.62 |
| *He* | 0.5 | 0.48 | 0.5 | 0.46 | 0.5 | 0.52 | 0.47 | 0.46 | 0.51 |
| *NULL* | 0.00 | 0.06 | 0.00 | 0.00 | 0.00 | 0.00 | 0.00 | 0.00 | 0.00 |
| *F_IS_* | -0.61 | 0.17 | -0.33 | -0.18 | -0.17 | -0.16 | -0.07 | -0.18 | -0.60 |
|  | *POCC1* | | | | | | | | |
| N | 20 | 6 | 6 | 11 | 14 | 15 | 9 | 11 | 92 |
| *NA* | 2 | 2 | 2 | 2 | 2 | 2 | 2 | 2 | 2 |
| *AR* | 1.94 | 1.66 | 1.97 | 1.77 | 1.99 | 1.87 | 1.98 | 1.95 | 1.89 |
| *Ho* | 0.45 | 0.20 | 0.33 | 0.27 | 0.58 | 0.33 | 0.50 | 0.36 | 0.38 |
| *He* | 0.35 | 0.18 | 0.44 | 0.24 | 0.41 | 0.28 | 0.38 | 0.40 | 0.34 |
| *NULL* | 0.00 | 0.00 | 0.08 | 0.00 | 0.00 | 0.00 | 0.00 | 0.03 | 0.00 |
| *F_IS_* | -0.27 | 0.00 | 0.00 | -0.11 | -0.38 | -0.17 | -0.23 | 0.13 | -0.17 |

**Appendix S2.4** Microsatellite locus genetic diversity measures for *P. stellata*: *N* – number of individuals; *NA* – number of alleles; *AR* – allelic richness; *HO* – observed heterozygosity; *HE* – expected heterozygosity; *NULL* – null allele frequency; *F_IS_* – inbreeding coefficient. *Significant deviations to Hardy–Weinberg equilibrium after Benjamini-Hochberg(*p* < 0.05)

|  | Ngele | Oribi Gorge | Gomo | Baziya | | Manubi | | Kubusi | | Fort Fordyce | | Alexandria | | The Island | | Overall |
| --- | --- | --- | --- | --- | --- | --- | --- | --- | --- | --- | --- | --- | --- | --- | --- | --- |
|  | *WBSW2* | | | | | | | | | | | | | | | |
| *N* | 30 | 13 | 26 | 15 | 28 | | 26 | | 39 | | 17 | | 6 | | 200 | |
| *NA* | 2 | 2 | 2 | 2 | 2 | | 2 | | 2 | | 2 | | 2 | | 2 | |
| *AR* | 1.98 | 2.00 | 2.00 | 1.98 | 2.00 | | 2.00 | | 2.00 | | 1.70 | | 1.68 | | 1.93 | |
| *Ho* | 0.37 | 0.58 | 0.56 | 0.33 | 0.43 | | 0.46 | | 0.45 | | 0.19 | | 0.17 | | 0.39 | |
| *He* | 0.43 | 0.47 | 0.49 | 0.42 | 0.50 | | 0.43 | | 0.49 | | 0.17 | | 0.15 | | 0.39 | |
| *NULL* | 0.04 | 0.00 | 0.00 | 0.00 | 0.00 | | 0.00 | | 0.00 | | 0.00 | | 0.00 | | 0.03 | |
| *F_IS_* | 0.17 | -0.20 | -0.12 | 0.24 | 0.16 | | -0.06 | | 0.10 | | -0.07 | | 0.00 | | 0.05 | |
|  | *Mcu4* | | | | | | | | | | | | | | | |
| *N* | 30 | 13 | 26 | 15 | 28 | | 26 | | 39 | | 17 | | 6 | | 200 | |
| *NA* | 4 | 3 | 4 | 4 | 3 | | 4 | | 3 | | 2 | | 2 | | 5 | |
| *AR* | 2.75 | 2.81 | 2.85 | 2.65 | 2.61 | | 2.48 | | 2.56 | | 2.00 | | 1.65 | | 2.48 | |
| *Ho* | 0.53 | 0.43 | 0.48 | 0.53 | 0.54 | | 0.33 | | 0.42 | | 0.62 | | 0.17 | | 0.45 | |
| *He* | 0.55 | 0.64 | 0.59 | 0.55 | 0.54 | | 0.46 | | 0.52 | | 0.49 | | 0.15 | | 0.50 | |
| *NULL* | 0.00 | 0.09 | 0.05 | 0.00 | 0.00 | | 0.05 | | 0.04 | | 0.00 | | 0.00 | | 0.04 | |
| *F_IS_* | 0.05 | 0.51 | 0.20 | 0.07 | 0.03 | | 0.51 | | 0.30 | | -0.24 | | 0.00 | | 0.18 | |
|  | *CNA142* | | | | | | | | | | | | | | | |
| *N* | 30 | 13 | 26 | 15 | 28 | | 26 | | 39 | | 17 | | 6 | | 200 | |
| *NA* | 10 | 7 | 7 | 8 | 10 | | 11 | | 9 | | 9 | | 5 | | 12 | |
| *AR* | 5.18 | 4.78 | 5.02 | 5.26 | 5.52 | | 6.42 | | 5.64 | | 5.79 | | 4.44 | | 5.34 | |
| *Ho* | 0.80 | 0.67 | 0.76 | 0.87 | 0.79 | | 0.85 | | 0.82 | | 0.75 | | 0.83 | | 0.79 | |
| *He* | 0.75 | 0.72 | 0.76 | 0.78 | 0.81 | | 0.86 | | 0.83 | | 0.84 | | 0.76 | | 0.79 | |
| *NULL* | 0.00 | 0.00 | 0.01 | 0.00 | 0.00 | | 0.00 | | 0.00 | | 0.04 | | 0.00 | | 0.01 | |
| *F_IS_* | -0.05 | 0.12 | 0.02 | -0.07 | 0.04 | | 0.04 | | 0.03 | | 0.14 | | 0.00 | | 0.03 | |
|  | *WBSW9* | | | | | | | | | | | | | | | |
| *N* | 30 | 13 | 26 | 15 | 28 | | 26 | | 39 | | 17 | | 6 | | 200 | |
| *NA* | 3 | 3 | 3 | 4 | 3 | | 2 | | 2 | | 2 | | 2 | | 6 | |
| *AR* | 2.17 | 2.37 | 2.19 | 2.56 | 2.07 | | 1.98 | | 1.95 | | 2.00 | | 1.99 | | 2.14 | |
| *Ho* | 0.47 | 0.33 | 0.48 | 0.47 | 0.25 | | 0.42 | | 0.37 | | 0.56 | | 0.50 | | 0.43 | |
| *He* | 0.50 | 0.43 | 0.41 | 0.38 | 0.33 | | 0.41 | | 0.33 | | 0.45 | | 0.38 | | 0.40 | |
| *NULL* | 0.02 | 0.05 | 0.00 | 0.00 | 0.10 | | 0.00 | | 0.00 | | 0.00 | | 0.00 | | 0.02 | |
| *F_IS_* | 0.09 | 0.27 | -0.14 | -0.19 | 0.25 | | -0.01 | | -0.10 | | -0.22 | | -0.25 | | -0.01 | |

**Appendix S2.4** (continued)

|  | Ngele | Oribi Gorge | Gomo | Baziya | | Manubi | | Kubusi | | Fort Fordyce | | Alexandria | | The Island | | Overall |
| --- | --- | --- | --- | --- | --- | --- | --- | --- | --- | --- | --- | --- | --- | --- | --- | --- |
|  | *CNA162* | | | | | | | | | | | | | | | |
| *N* | 30 | 13 | 26 | 15 | 28 | | 26 | | 39 | | 17 | | 6 | | 200 | |
| *NA* | 6 | 6 | 7 | 7 | 6 | | 6 | | 7 | | 4 | | 6 | | 13 | |
| *AR* | 4.99 | 4.87 | 4.80 | 4.96 | 4.75 | | 4.28 | | 4.74 | | 2.84 | | 4.86 | | 4.57 | |
| *Ho* | 0.73 | 0.75 | 0.84 | 0.73 | 0.61 | | 0.69 | | 0.66 | | 0.62 | | 1.00 | | 0.74 | |
| *He* | 0.80 | 0.80 | 0.78 | 0.78 | 0.78 | | 0.72 | | 0.78 | | 0.58 | | 0.78 | | 0.76 | |
| *NULL* | 0.02 | 0.01 | 0.00 | 0.00 | 0.09 | | 0.01 | | 0.07 | | 0.00 | | 0.00 | | 0.03 | |
| *F_IS_* | 0.10 | 0.10 | -0.05 | 0.10 | 0.24 | | 0.06 | | 0.17 | | -0.05 | | -0.20 | | 0.09 | |
|  | *CACA3* | | | | | | | | | | | | | | | |
| *N* | 30 | 13 | 26 | 15 | 28 | | 26 | | 39 | | 17 | | 6 | | 200 | |
| *NA* | 7 | 5 | 7 | 5 | 6 | | 7 | | 5 | | 4 | | 4 | | 10 | |
| *AR* | 3.74 | 3.50 | 3.87 | 3.20 | 3.82 | | 3.90 | | 3.28 | | 2.61 | | 3.26 | | 3.46 | |
| *Ho* | 0.57 | 0.67 | 0.76 | 0.53 | 0.61 | | 0.62 | | 0.58 | | 0.56 | | 0.50 | | 0.60 | |
| *He* | 0.56 | 0.63 | 0.64 | 0.51 | 0.67 | | 0.64 | | 0.54 | | 0.53 | | 0.51 | | 0.58 | |
| *NULL* | 0.00 | 0.00 | 0.00 | 0.00 | 0.00 | | 0.00 | | 0.00 | | 0.05 | | 0.00 | | 0.00 | |
| *F_IS_* | 0.01 | -0.02 | -0.17 | -0.01 | 0.11 | | 0.05 | | -0.07 | | -0.04 | | 0.12 | | -0.01 | |
|  | *CACA27* | | | | | | | | | | | | | | | |
| *N* | 30 | 13 | 26 | 15 | 28 | | 26 | | 39 | | 17 | | 6 | | 200 | |
| *NA* | 14 | 8 | 12 | 10 | 12 | | 11 | | 12 | | 9 | | 10 | | 20 | |
| *AR* | 7.68 | 5.73 | 6.89 | 6.21 | 7.06 | | 6.51 | | 6.51 | | 6.32 | | 7.13 | | 6.67 | |
| *Ho* | 0.97 | 0.92 | 0.88 | 0.93 | 0.89 | | 0.85 | | 0.84 | | 0.94 | | 1.00 | | 0.91 | |
| *He* | 0.91 | 0.82 | 0.89 | 0.85 | 0.89 | | 0.87 | | 0.85 | | 0.86 | | 0.89 | | 0.87 | |
| *NULL* | 0.00 | 0.00 | 0.01 | 0.00 | 0.00 | | 0.00 | | 0.00 | | 0.00 | | 0.00 | | 0.00 | |
| *F_IS_* | -0.05 | -0.07 | 0.03 | -0.06 | 0.01 | | 0.04 | | 0.03 | | -0.06 | | -0.03 | | -0.01 | |
|  | *Gf06* | | | | | | | | | | | | | | | |
| *N* | 30 | 13 | 26 | 15 | 28 | | 26 | | 39 | | 17 | | 6 | | 200 | |
| *NA* | 7 | 5 | 7 | 7 | 8 | | 7 | | 7 | | 6 | | 4 | | 20 | |
| *AR* | 4.84 | 3.96 | 4.61 | 5.34 | 4.39 | | 4.39 | | 4.69 | | 4.72 | | 3.88 | | 4.54 | |
| *Ho* | 0.90 | 0.75 | 0.88 | 0.93 | 0.79 | | 0.73 | | 0.89 | | 1.00 | | 1.00 | | 0.87 | |
| *He* | 0.79 | 0.73 | 0.76 | 0.81 | 0.73 | | 0.73 | | 0.78 | | 0.77 | | 0.74 | | 0.76 | |
| *NULL* | 0.00 | 0.00 | 0.00 | 0.00 | 0.00 | | 0.00 | | 0.00 | | 0.00 | | 0.00 | | 0.00 | |
| *F_IS_* | -0.12 | 0.01 | -0.13 | -0.12 | -0.06 | | 0.02 | | -0.14 | | -0.26 | | -0.28 | | -0.11 | |

# Appendix S3: Hardy-Weinberg equilibrium

| **Appendix 3.1** Hardy-Weinberg equilibrium of locus at the forest level in *Batis capensis.* ******* denotes significant HWE after Benjamini-Hochberg correction. | | | |  |
| --- | --- | --- | --- | --- |
|  |  |  |  |  |
| **Forest** | **Locus** | ***p* value** | **Corrected *p* value** |  |
| Ngele | BMI16 | 1.0000 | 1.0000 |  |
| Ngele | BMI17 | 1.0000 | 1.0000 |  |
| Ngele | BMI22 | 1.0000 | 1.0000 |  |
| Ngele | BMI44 | 1.0000 | 1.0000 |  |
| Ngele | BMI96 | 1.0000 | 1.0000 |  |
| Ngele | BMI32 | 0.2398 | 0.6772 |  |
| Ngele | BMI98 | 0.6917 | 1.0000 |  |
| Ngele | BMI71 | 1.0000 | 1.0000 |  |
| Oribi Gorge | BMI16 | 0.6188 | 1.0000 |  |
| Oribi Gorge | BMI17 | 1.0000 | 1.0000 |  |
| Oribi Gorge | BMI22 | 0.4705 | 1.0000 |  |
| Oribi Gorge | BMI44 | 1.0000 | 1.0000 |  |
| Oribi Gorge | BMI96 | 0.6226 | 1.0000 |  |
| Oribi Gorge | BMI32 | 0.1504 | 0.4853 |  |
| Oribi Gorge | BMI98 | 0.3478 | 0.8516 |  |
| Oribi Gorge | BMI71 | 0.1114 | 0.4852 |  |
| Gomo | BMI16 | 0.0315 | 0.1675 |  |
| Gomo | BMI17 | 0.7079 | 1.0000 |  |
| Gomo | BMI22 | 0.3672 | 0.8558 |  |
| Gomo | BMI44 | 0.0330 | 0.1675 |  |
| Gomo | BMI96 | 0.6367 | 1.0000 |  |
| Gomo | BMI32 | 0.1162 | 0.4852 |  |
| Gomo | BMI98 | 0.1319 | 0.4853 |  |
| Gomo | BMI71 | 0.0023 | 0.1762 |  |
| Nqadu | BMI16 | 1.0000 | 1.0000 |  |
| Nqadu | BMI17 | 0.8127 | 1.0000 |  |
| Nqadu | BMI22 | 0.6212 | 1.0000 |  |
| Nqadu | BMI44 | 0.2837 | 0.7459 |  |
| Nqadu | BMI96 | 1.0000 | 1.0000 |  |
| Nqadu | BMI32 | 0.2480 | 0.6772 |  |
| Nqadu | BMI98 | 0.1480 | 0.4853 |  |
| **Nqadu** | **BMI71** | **0.0003** | **0.0040*** |  |
| Baziya | BMI16 | 0.0315 | 0.1675 |  |
| Baziya | BMI17 | 0.7511 | 1.0000 |  |
| Baziya | BMI22 | 0.7744 | 1.0000 |  |
| Baziya | BMI44 | 0.2184 | 0.6462 |  |
| Baziya | BMI96 | 1.0000 | 1.0000 |  |
| Baziya | BMI32 | 0.1317 | 0.4853 |  |
| Baziya | BMI98 | 0.6305 | 1.0000 |  |
| Baziya | BMI71 | 0.0648 | 0.2269 |  |
| Manubi | BMI16 | 0.0315 | 0.1675 |  |
| Manubi | BMI17 | 0.5378 | 1.0000 |  |
| Manubi | BMI22 | 1.0000 | 1.0000 |  |
| Manubi | BMI44 | 1.0000 | 1.0000 |  |
| Manubi | BMI96 | 0.5516 | 1.0000 |  |
| Manubi | BMI32 | 0.9640 | 1.0000 |  |
| Manubi | BMI98 | 0.0315 | 0.1675 |  |
| Manubi | BMI71 | 0.3737 | 0.8558 |  |
| Kubusi | BMI16 | 0.9847 | 1.0000 |  |
| Kubusi | BMI17 | 0.9896 | 1.0000 |  |
| Kubusi | BMI22 | 0.5223 | 1.0000 |  |
| Kubusi | BMI44 | 0.9004 | 1.0000 |  |
| Kubusi | BMI96 | 0.1486 | 0.4853 |  |
| Kubusi | BMI32 | 0.7410 | 1.0000 |  |
| Kubusi | BMI98 | 0.0542 | 0.2567 |  |
| Kubusi | BMI71 | 0.0134 | 0.0792 |  |
| Fort Fordyce | BMI16 | 0.4433 | 0.9837 |  |
| Fort Fordyce | BMI17 | 0.9285 | 1.0000 |  |
| Fort Fordyce | BMI22 | 1.0000 | 1.0000 |  |
| Fort Fordyce | BMI44 | 0.9855 | 1.0000 |  |
| Fort Fordyce | BMI96 | 0.0542 | 0.2567 |  |
| Fort Fordyce | BMI32 | 0.0542 | 0.2567 |  |
| Fort Fordyce | BMI98 | 0.0251 | 0.1980 |  |
| Fort Fordyce | BMI71 | 0.0134 | 0.0792 |  |
| The Island | BMI16 | 0.8794 | 1.0000 |  |
| The Island | BMI17 | 0.2064 | 0.6371 |  |
| The Island | BMI22 | 1.0000 | 1.0000 |  |
| The Island | BMI44 | 0.2950 | 0.7481 |  |
| The Island | BMI96 | 0.9546 | 1.0000 |  |
| The Island | BMI32 | 0.7695 | 1.0000 |  |
| **The Island** | **BMI98** | **0.0025** | **0.0198*** |  |
| The Island | BMI71 | 0.3515 | 0.7989 |  |

| **Appendix 3.2** Hardy-Weinberg equilibrium of locus at the forest level in *Cossypha dichroa*. ******* denotes significant HWE after Benjamini-Hochberg correction. | | | |  |
| --- | --- | --- | --- | --- |
|  |  |  |  |  |
| **Forest** | **Locus** | ***p* value** | **Corrected *p* value** |  |
| Ngele | WBSW9 | 1.0000 | 1.0000 |  |
| Ngele | CNA130 | 0.1208 | 0.8476 |  |
| Ngele | CNA109 | 0.3043 | 0.9999 |  |
| Ngele | CNA99 | 0.6514 | 1.0000 |  |
| Ngele | CNA142 | 0.3444 | 0.9999 |  |
| Ngele | CNA113 | 0.7219 | 1.0000 |  |
| Ngele | CACA3 | 1.0000 | 1.0000 |  |
| Ngele | CNA180 | 0.5166 | 1.0000 |  |
| Oribi Gorge | WBSW9 | 1.0000 | 1.0000 |  |
| Oribi Gorge | CNA130 | 1.0000 | 1.0000 |  |
| Oribi Gorge | CNA109 | 0.4255 | 0.9999 |  |
| Oribi Gorge | CNA99 | 0.4285 | 0.9999 |  |
| Oribi Gorge | CNA142 | 1.0000 | 1.0000 |  |
| Oribi Gorge | CNA113 | 0.1422 | 0.8846 |  |
| Oribi Gorge | CACA3 | 1.0000 | 1.0000 |  |
| Oribi Gorge | CNA180 | 1.0000 | 1.0000 |  |
| Baziya | WBSW9 | 1.0000 | 1.0000 |  |
| Baziya | CNA130 | 0.0361 | 0.8476 |  |
| Baziya | CNA109 | 0.3358 | 0.9999 |  |
| Baziya | CNA99 | 0.4487 | 1.0000 |  |
| Baziya | CNA142 | 0.1130 | 0.8476 |  |
| Baziya | CNA113 | 0.9304 | 1.0000 |  |
| Baziya | CACA3 | 0.8424 | 1.0000 |  |
| Baziya | CNA180 | 0.5851 | 1.0000 |  |
| Manubi | WBSW9 | 0.2172 | 0.9999 |  |
| Manubi | CNA130 | 0.7330 | 1.0000 |  |
| Manubi | CNA109 | 0.1936 | 0.9937 |  |
| Manubi | CNA99 | 0.3768 | 0.9999 |  |
| Manubi | CNA142 | 0.5378 | 1.0000 |  |
| Manubi | CNA113 | 0.5671 | 1.0000 |  |
| Manubi | CACA3 | 0.3941 | 0.9999 |  |
| Manubi | CNA180 | 0.0779 | 0.8476 |  |
| Kubusi | WBSW9 | 0.1952 | 0.9937 |  |
| Kubusi | CNA130 | 0.9555 | 1.0000 |  |
| Kubusi | CNA109 | 0.8233 | 1.0000 |  |
| Kubusi | CNA99 | 0.0468 | 0.8476 |  |
| Kubusi | CNA142 | 0.1211 | 0.8476 |  |
| Kubusi | CNA113 | 0.6795 | 1.0000 |  |
| Kubusi | CACA3 | 0.3938 | 0.9999 |  |
| Kubusi | CNA180 | 0.0180 | 0.8476 |  |
| Fort Fordyce | WBSW9 | 0.4103 | 0.9999 |  |
| Fort Fordyce | CNA130 | 0.7448 | 1.0000 |  |
| Fort Fordyce | CNA109 | 0.6476 | 1.0000 |  |
| Fort Fordyce | CNA99 | 0.8983 | 1.0000 |  |
| Fort Fordyce | CNA142 | 0.3133 | 0.9999 |  |
| Fort Fordyce | CNA113 | 0.9523 | 1.0000 |  |
| Fort Fordyce | CACA3 | 1.0000 | 1.0000 |  |
| Fort Fordyce | CNA180 | 0.0622 | 0.8476 |  |
| Alexandria | WBSW9 | 0.7091 | 1.0000 |  |
| Alexandria | CNA130 | 0.7910 | 1.0000 |  |
| Alexandria | CNA109 | 0.2608 | 0.9999 |  |
| Alexandria | CNA99 | 0.5824 | 1.0000 |  |
| Alexandria | CNA142 | 0.3045 | 0.9999 |  |
| Alexandria | CNA113 | 1.0000 | 1.0000 |  |
| Alexandria | CACA3 | 1.0000 | 1.0000 |  |
| Alexandria | CNA180 | 1.0000 | 1.0000 |  |

| **Appendix 3.3** Hardy-Weinberg equilibrium of locus at the forest level in *Phylloscopus ruficapilla*. ******* denotes significant HWE after Benjamini-Hochberg correction. | | | |  |
| --- | --- | --- | --- | --- |
|  |  |  |  |  |
| **Forest** | **Locus** | ***p* value** | **Corrected *p* value** |  |
| Pat43 | Ngele | 0.3280 | 1.0000 |  |
| MLSP4 | Ngele | 1.0000 | 1.0000 |  |
| Dpu16 | Ngele | 1.0000 | 1.0000 |  |
| Cu02 | Ngele | 0.7870 | 1.0000 |  |
| POCC9 | Ngele | 1.0000 | 1.0000 |  |
| POCC6 | Ngele | 0.3536 | 1.0000 |  |
| POCC7 | Ngele | 0.0220 | 0.4626 |  |
| POCC1 | Ngele | 0.5278 | 1.0000 |  |
| Pat43 | Oribi Gorge | 1.0000 | 1.0000 |  |
| MLSP4 | Oribi Gorge | 1.0000 | 1.0000 |  |
| Dpu16 | Oribi Gorge | 1.0000 | 1.0000 |  |
| Cu02 | Oribi Gorge | 1.0000 | 1.0000 |  |
| POCC9 | Oribi Gorge | 0.0164 | 0.4626 |  |
| POCC6 | Oribi Gorge | 1.0000 | 1.0000 |  |
| POCC7 | Oribi Gorge | 1.0000 | 1.0000 |  |
| POCC1 | Oribi Gorge | 1.0000 | 1.0000 |  |
| Pat43 | Mbotyi | 0.4292 | 1.0000 |  |
| MLSP4 | Mbotyi | 1.0000 | 1.0000 |  |
| Dpu16 | Mbotyi | 1.0000 | 1.0000 |  |
| Cu02 | Mbotyi | 0.4294 | 1.0000 |  |
| POCC9 | Mbotyi | 0.4304 | 1.0000 |  |
| POCC6 | Mbotyi | 0.4286 | 1.0000 |  |
| POCC7 | Mbotyi | 1.0000 | 1.0000 |  |
| POCC1 | Mbotyi | 1.0000 | 1.0000 |  |
| Pat43 | Gomo | 1.0000 | 1.0000 |  |
| MLSP4 | Gomo | 1.0000 | 1.0000 |  |
| Dpu16 | Gomo | 1.0000 | 1.0000 |  |
| Cu02 | Gomo | 0.2572 | 1.0000 |  |
| POCC9 | Gomo | 1.0000 | 1.0000 |  |
| POCC6 | Gomo | 1.0000 | 1.0000 |  |
| POCC7 | Gomo | 1.0000 | 1.0000 |  |
| POCC1 | Gomo | 1.0000 | 1.0000 |  |
| Pat43 | Baziya | 0.4043 | 1.0000 |  |
| MLSP4 | Baziya | 0.0884 | 1.0000 |  |
| Dpu16 | Baziya | 1.0000 | 1.0000 |  |
| Cu02 | Baziya | 1.0000 | 1.0000 |  |
| POCC9 | Baziya | 1.0000 | 1.0000 |  |
| POCC6 | Baziya | 0.4033 | 1.0000 |  |
| POCC7 | Baziya | 1.0000 | 1.0000 |  |
| POCC1 | Baziya | 0.4875 | 1.0000 |  |
| Pat43 | Manubi | 1.0000 | 1.0000 |  |
| MLSP4 | Manubi | 1.0000 | 1.0000 |  |
| Dpu16 | Manubi | 1.0000 | 1.0000 |  |
| Cu02 | Manubi | 1.0000 | 1.0000 |  |
| POCC9 | Manubi | 0.0041 | 0.2570 |  |
| POCC6 | Manubi | 0.5394 | 1.0000 |  |
| POCC7 | Manubi | 1.0000 | 1.0000 |  |
| POCC1 | Manubi | 1.0000 | 1.0000 |  |
| Pat43 | Kubusi | 1.0000 | 1.0000 |  |
| MLSP4 | Kubusi | 1.0000 | 1.0000 |  |
| Dpu16 | Kubusi | 1.0000 | 1.0000 |  |
| Cu02 | Kubusi | 1.0000 | 1.0000 |  |
| POCC9 | Kubusi | 1.0000 | 1.0000 |  |
| POCC6 | Kubusi | 1.0000 | 1.0000 |  |
| POCC7 | Kubusi | 1.0000 | 1.0000 |  |
| POCC1 | Kubusi | 1.0000 | 1.0000 |  |
| Pat43 | Fort Fordyce | 1.0000 | 1.0000 |  |
| MLSP4 | Fort Fordyce | 1.0000 | 1.0000 |  |
| Dpu16 | Fort Fordyce | 1.0000 | 1.0000 |  |
| Cu02 | Fort Fordyce | 0.5545 | 1.0000 |  |
| POCC9 | Fort Fordyce | 0.4385 | 1.0000 |  |
| POCC6 | Fort Fordyce | 1.0000 | 1.0000 |  |
| POCC7 | Fort Fordyce | 1.0000 | 1.0000 |  |
| POCC1 | Fort Fordyce | 1.0000 | 1.0000 |  |

| **Appendix 3.4** Hardy-Weinberg equilibrium of locus at the forest level in *Pogonocichla stellata*. ******* denotes significant HWE after Benjamini-Hochberg correction. | | | |  |
| --- | --- | --- | --- | --- |
|  |  |  |  |  |
| **Forest** | **Locus** | **p** | **corrected p** |  |
| Ngele | WBSW2 | 0.4123 | 0.9575 |  |
| Ngele | MCU4 | 0.2725 | 0.8501 |  |
| Ngele | CNA142 | 0.7878 | 1.0000 |  |
| Ngele | WBSW9 | 0.8337 | 1.0000 |  |
| Ngele | CNA162 | 0.2327 | 0.8471 |  |
| Ngele | CACA3 | 0.1847 | 0.8471 |  |
| Ngele | CACA27 | 0.9069 | 1.0000 |  |
| Ngele | GF06 | 0.1517 | 0.8400 |  |
| Oribi Gorge | WBSW2 | 0.5933 | 1.0000 |  |
| Oribi Gorge | MCU4 | 0.0237 | 0.2434 |  |
| Oribi Gorge | CNA142 | 0.1338 | 0.8400 |  |
| Oribi Gorge | WBSW9 | 0.1957 | 0.8471 |  |
| Oribi Gorge | CNA162 | 0.9108 | 1.0000 |  |
| Oribi Gorge | CACA3 | 0.3329 | 0.8561 |  |
| Oribi Gorge | CACA27 | 0.8168 | 1.0000 |  |
| Oribi Gorge | GF06 | 0.8364 | 1.0000 |  |
| Gomo | WBSW2 | 0.6906 | 1.0000 |  |
| Gomo | MCU4 | 0.3834 | 0.9519 |  |
| Gomo | CNA142 | 0.2035 | 0.8471 |  |
| Gomo | WBSW9 | 0.7345 | 1.0000 |  |
| Gomo | CNA162 | 0.2437 | 0.8471 |  |
| Gomo | CACA3 | 0.4618 | 0.9869 |  |
| Gomo | CACA27 | 0.2516 | 0.8471 |  |
| Gomo | GF06 | 0.3997 | 0.9575 |  |
| Baziya | WBSW2 | 0.5396 | 1.0000 |  |
| Baziya | MCU4 | 1.0000 | 1.0000 |  |
| Baziya | CNA142 | 0.8208 | 1.0000 |  |
| Baziya | WBSW9 | 1.0000 | 1.0000 |  |
| Baziya | CNA162 | 0.0320 | 0.2877 |  |
| Baziya | CACA3 | 0.4890 | 1.0000 |  |
| Baziya | CACA27 | 0.9154 | 1.0000 |  |
| Baziya | GF06 | 0.9630 | 1.0000 |  |
| Manubi | WBSW2 | 0.4660 | 0.9869 |  |
| Manubi | MCU4 | 0.4486 | 0.9869 |  |
| Manubi | CNA142 | 0.7671 | 1.0000 |  |
| Manubi | WBSW9 | 0.0181 | 0.2166 |  |
| Manubi | CNA162 | 0.0058 | 0.1397 |  |
| Manubi | CACA3 | 0.1767 | 0.8471 |  |
| Manubi | CACA27 | 0.2588 | 0.8471 |  |
| Manubi | GF06 | 0.8096 | 1.0000 |  |
| Kubusi | WBSW2 | 1.0000 | 1.0000 |  |
| Kubusi | MCU4 | 0.0048 | 0.1397 |  |
| Kubusi | CNA142 | 0.5744 | 1.0000 |  |
| Kubusi | WBSW9 | 1.0000 | 1.0000 |  |
| Kubusi | CNA162 | 0.3058 | 0.8532 |  |
| Kubusi | CACA3 | 0.7195 | 1.0000 |  |
| Kubusi | CACA27 | 0.3195 | 0.8532 |  |
| Kubusi | GF06 | 0.5542 | 1.0000 |  |
| Fort Fordyce | WBSW2 | 0.7400 | 1.0000 |  |
| Fort Fordyce | MCU4 | 0.0111 | 0.1698 |  |
| Fort Fordyce | CNA142 | 0.0058 | 0.1397 |  |
| Fort Fordyce | WBSW9 | 1.0000 | 1.0000 |  |
| Fort Fordyce | CNA162 | 0.0926 | 0.6670 |  |
| Fort Fordyce | CACA3 | 0.5763 | 1.0000 |  |
| Fort Fordyce | CACA27 | 0.3199 | 0.8532 |  |
| Fort Fordyce | GF06 | 0.2195 | 0.8471 |  |
| Alexandria | WBSW2 | 1.0000 | 1.0000 |  |
| Alexandria | MCU4 | 0.6088 | 1.0000 |  |
| Alexandria | CNA142 | 0.0654 | 0.5233 |  |
| Alexandria | WBSW9 | 0.5923 | 1.0000 |  |
| Alexandria | CNA162 | 0.8972 | 1.0000 |  |
| Alexandria | CACA3 | 0.0118 | 0.1698 |  |
| Alexandria | CACA27 | 0.9188 | 1.0000 |  |
| Alexandria | GF06 | 0.2834 | 0.8501 |  |
| The Island | WBSW2 | 1.0000 | 1.0000 |  |
| The Island | MCU4 | 1.0000 | 1.0000 |  |
| The Island | CNA142 | 0.9447 | 1.0000 |  |
| The Island | WBSW9 | 1.0000 | 1.0000 |  |
| The Island | CNA162 | 1.0000 | 1.0000 |  |
| The Island | CACA3 | 0.5185 | 1.0000 |  |
| The Island | CACA27 | 1.0000 | 1.0000 |  |
| The Island | GF06 | 0.1500 | 0.8400 |  |
